# When Light Challenges Heat: Mechanistic Insights Into a Reaction Competing With Cadogan Cyclisation in Nitro‐Perylenediimides

**DOI:** 10.1002/chem.70970

**Published:** 2026-04-09

**Authors:** Manuel Pedrón Laserna, Ilaria Ciofini, Piétrick Hudhomme

**Affiliations:** ^1^ Chimie Paris Tech CNRS Institute of Chemistry for Life and Health Sciences PSL University Paris France; ^2^ Institute of Biocomputation and Physics of Complex Systems (BIFI) University of Zaragoza Zaragoza Spain; ^3^ Univ Angers CNRS MOLTECH‐Anjou SFR MATRIX Angers France

## Abstract

Visible‐light‐driven transformations have emerged as powerful and sustainable tools in modern organic synthesis. However, the intrinsic photochemical reactivity of polycyclic aromatic hydrocarbons (PAHs) remains underexplored. Among π‐conjugated chromophores, perylenediimides (PDIs) combine exceptional photostability, strong visible‐light absorption, and rich redox properties, yet their light‐induced chemical transformations are still poorly understood. Herein, we report an unprecedented divergence between thermal and photochemical reactivity in the reaction of bay‐nitrated PDIs (PDI‐NO_2_) with triphenylphosphine. While thermal activation promotes a classical Cadogan‐type reductive cyclization to afford *N*‐annulated PDI carbazole, visible‐light irradiation redirects the reaction toward a previously unobserved pathway, yielding a bay‐functionalized 1‐(iminophosphorane)‐12‐hydroxy PDI derivative in excellent yield. Experimental studies reveal a strong wavelength dependence, with blue light dominating the photochemical transformation. Notably, the initial nitro‐to‐nitroso conversion is not phosphine‐mediated but arises from the strong reducing power of photoexcited PDI‐NO_2_. Combined experimental and theoretical investigations demonstrate that light irradiation reshapes the reaction landscape by enabling access to charge‐transfer and π–π* excited states, involving the population of an asymmetric unoccupied orbital localized on the nitroso moiety, thereby unlocking a phosphine‐addition pathway inaccessible under thermal conditions. These findings establish orbital‐selective excitation as a general design principle for exploiting visible light to control reaction pathways in π‐conjugated chromophores.

## Graphical abstract

Visible‐light irradiation of nitro‐perylenediimide in the presence of triphenylphosphine reveals an unexpected pathway competing with the classical Cadogan cyclization. Mechanistic investigations uncover the rapid and selective formation of bay‐functionalized 1‐iminophosphorane‐12‐hydroxy perylenediimide derivatives.

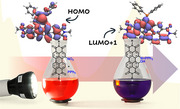

## 1 Introduction

Modern organic chemistry has increasingly focused on the development of sustainable, efficient, and selective synthetic strategies [1, 2] Among these, visible‐light‐driven reactions have emerged as powerful tools for promoting highly selective chemical transformations under mild, environmentally friendly conditions, while offering excellent functional group tolerance [3, 4]. Despite these advances, their application to the functionalization of polycyclic aromatic hydrocarbons (PAHs) remains comparatively limited [5, 6]. Within the broad family of photoactive π‐conjugated chromophores, perylenediimides (PDIs) occupy a prominent position owing to their exceptional photostability, strong visible‐light absorption, and reversible redox properties. These features have established PDIs as key molecular scaffolds in organic electronics [7, 8], photocatalysis [9, 10], and bio‐related chemistry [11, 12]. Beyond their widespread use as functional materials, PDIs also represent attractive platforms for exploring light‐induced chemical reactivity. In particular, bay‐region nitration provides a selective electrophilic functionalization strategy that not only represents an efficient alternative to classical brominated derivatives used in nucleophilic substitution reactions [13, 14, 15], but also introduces a nitro group that modulates the electronic properties and serves as a versatile intermediate for subsequent annulation reactions leading to π‐extended, nitrogen‐containing PDI architectures.

**SCHEME 1:**
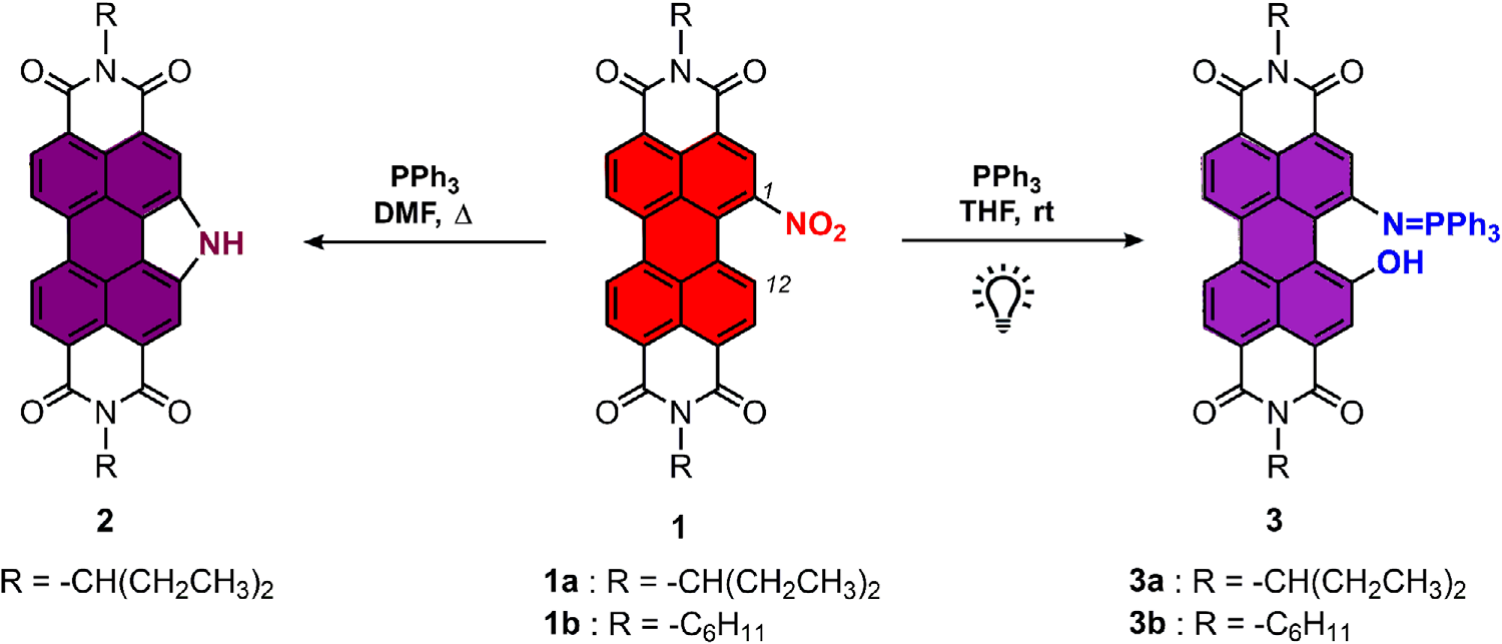
Reactivity of nitro‐PDI with triphenylphosphine under thermal or visible‐light‐driven conditions.

Cadogan [16, 17, 18], and modified Cadogan‐Sundberg [19, 20] reactions constitute well‐established methods for the synthesis of azaheterocycles from nitro‐ or nitroso‐substituted aromatics [21, 22, 23, 24]. These annulation processes are typically carried out using trialkylphosphite or trialkyl/triphenylphosphine reagents under harsh thermal conditions [25, 26, 27] although dioxomolybdenum(VI) catalysis has emerged as a milder alternative [28]. When applied to nitro‐PDI derivative, Cadogan‐type reductive cyclization provides efficient access to *N*‐annulated PDI semiconductors displaying remarkable n‐type charge‐transport behaviour [29, 30, 31], as well as, more recently, valuable photocatalytic activity [32, 33]. However, this transformation still requires elevated temperatures in the presence of triphenylphosphine (Scheme 1) [34].

While such thermal and catalytic protocols remain widely used, recent advances have opened the way to photochemical variants of the Cadogan reaction. Visible‐light‐driven Cadogan‐type cyclizations have been achieved using donor‐acceptor‐type cyanoarene photocatalysts, allowing transition‐metal‐free deoxygenation under blue LED irradiation (380‐500 nm) and providing a broad range of carbazoles and related nitrogen heterocycles with excellent functional group tolerance [35]. In the PDI series, an alternative route based on nucleophilic substitution of the nitro group by sodium azide, followed by photolysis of the resulting azido intermediate, afforded *N*‐annulated PDIs in quantitative yield under mild conditions [36]. Despite extensive photophysical studies on PDIs, the intrinsic chemical reactivity of PDIs under light irradiation remains comparatively underexplored. To the best of our knowledge, only a photoisomerization process converting nitroPDI into nitritoPDI in acetonitrile has been reported [37]. In our recent studies, we discovered that exposure of the nitro‐PDI derivative **1** to white LED irradiation in the presence of triphenylphosphine (PPh_3_) induces an unexpected reactivity. Instead of undergoing the Cadogan‐type cyclization to form the *N*‐annulated PDI **2**, the reaction selectively affords the bay‐functionalized 1‐(iminophosphorane)‐12‐hydroxy PDI derivative **3** in excellent yield (Scheme 1) [38].

This contrast between thermal and photochemical outcomes reveals a delicate competition between two mechanistic pathways, classical reductive cyclization or photoinduced phosphine addition, thereby offering new insight into the intrinsic photochemical reactivity of the PDI framework. In this work, an approach combining experimental insights and density functional theory (DFT) calculations is employed to elucidate the mechanism of this unprecedented light‐induced transformation. Theoretical analysis accounts for the divergence between Cadogan annulation and photoactivated phosphine addition, identifying the key factors governing regioselectivity and product distribution in nitro‐PDI derivatives. Overall, this study highlights a previously unexplored facet of PDI photoreactivity and outlines guiding principles for harnessing the full synthetic potential of PDIs under mild, visible‐light‐driven conditions.

## 2 Results and Discussion

**SCHEME 2:**
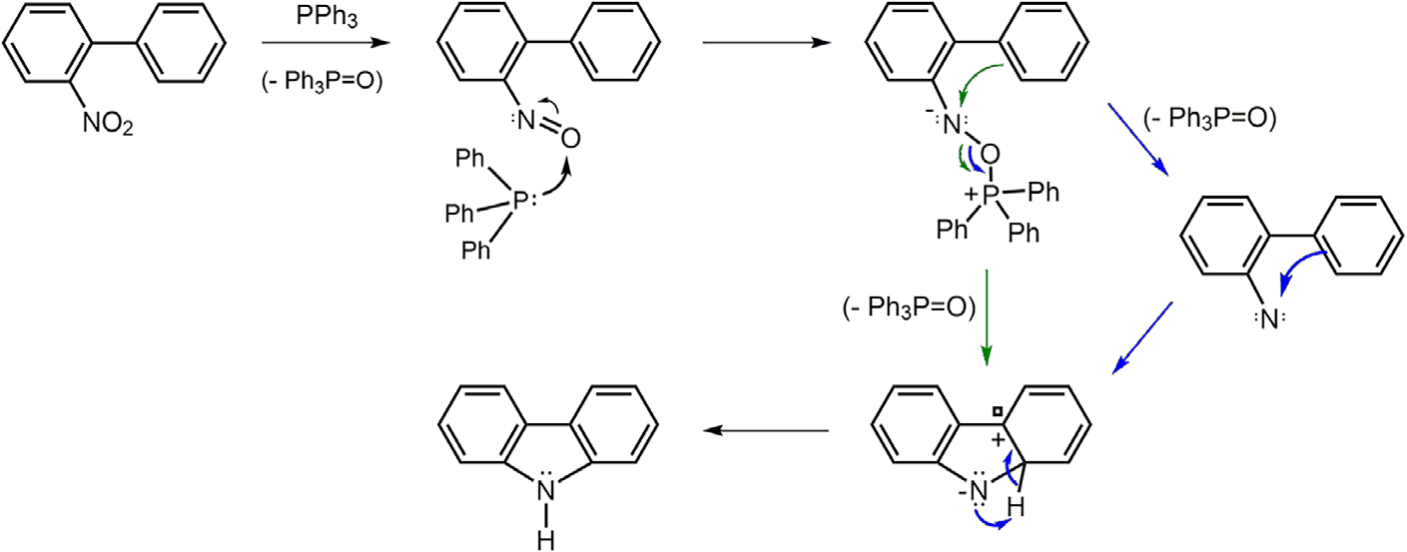
Proposed Cadogan reaction mechanism by A. W. Freeman et al. [26].

The Cadogan reaction is a widely used method for the synthesis of carbazoles from 2‐nitrobiphenyl or 2‐nitroso analogs, typically employing trialkyl phosphite or trialkyl/triphenylphosphine reagents. It is well established that the first step of the mechanism involves the phosphine‐ or phosphite‐mediated deoxygenation using one equivalent of PPh_3_ or P(OR)_3_ leading to the formation of the nitroso derivative (PDI‐NO) intermediate (Scheme 2) [21, 39, 40]. The reaction mechanism could then proceed through two distinct pathways, that is nitrene [41, 42] or nonnitrene pathway, as proposed by A.W. Freeman et al. [26].

**SCHEME 3:**
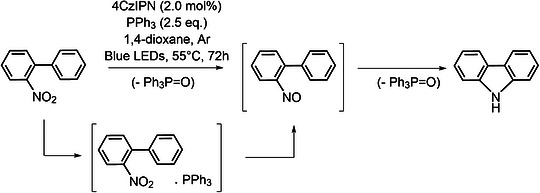
Reported photochemical conversion of 2‐nitrobiphenyl into carbazole [35].

The main visible‐light‐driven syntheses of carbazoles reported in the literature primarily rely on photocatalytic reactions of triarylamines and diarylamines [43, 44, 45], as well as on 2,2′‐diaminobiaryls [46]. Very recently, a highly efficient light‐promoted carbazole synthesis has been described starting from nitroarenes and Grignard reagents [47]. In this *one‐pot* process, the proposed in situ generated nitrosoarene intermediate undergoes thermal C─N bond formation, followed by a photoinduced aza‐6π electrocyclization. The photochemical step requires only purple light irradiation (390‐395 nm), without external catalysts or additives, providing a novel and step‐economical route to carbazoles. Conceptually related to this approach, a visible‐light‐driven photochemical Cadogan‐type reaction has recently been developed, enabling the conversion of 2‐nitrobiphenyl derivatives into carbazole analogues under blue LED irradiation in the presence of PPh_3_ and the photosensitizer 1,2,3,5‐*tetrakis*(carbazol‐9‐yl)‐4,6‐dicyanobenzene (4CzIPN) (Scheme 3) [35, 48]. DFT calculations revealed that 2‐nitrobiphenyl and PPh_3_ form a weak electron donor–acceptor complex, stabilized by Coulombic and π‐π interactions, with a slight endergonic character (ΔG = +2.2 kcal.mol^−1^). Upon energy transfer from the excited 4CzIPN, this complex is activated, triggering an oxygen transfer process that produces nitrosobiphenyl and triphenylphosphine oxide (Ph_3_P═O). Time‐dependent DFT (TD‐DFT) calculations indicate that the S_0_→S_2_ transition at 3.52 eV (352 nm) corresponds predominantly to a local excitation of the nitrobiphenyl moiety, explaining why this reaction proceeds under much milder conditions than conventional Cadogan cyclization. Overall, the transformation of 2‐nitrobiphenyl into carbazole requires two equivalents of PPh_3_.

Building on these recent developments, we investigated the applicability of visible‐light‐promoted Cadogan‐type reactions within the PDI series. Nitro‐PDI **1** [R = ─CH(C_4_H_9_)_2_] was reported to undergo reductive Cadogan cyclization in triethylphosphite at 130°C affording compound **2** in 56% yield [49]. When the reaction was performed with PPh_3_ in DMF at 150°C on the 1‐ethylpropyl‐substituted analogue [R = ─CH(C_2_H_5_)_2_], the yield increased to 67% (Scheme 1) [34] Considering recent advances in visible‐light‐promoted deoxygenation as an emerging strategy in green synthesis [50], we investigated whether this transformation could also proceed under photochemical conditions on nitro‐PDI. We discovered that irradiation of nitro‐PDI **1b** in the presence of PPh_3_ using an 18 W white LED source (400‐700 nm) induces an unexpected transformation. Instead of undergoing the expected Cadogan‐type cyclization to form the *N*‐annulated PDI **2**, the reaction rapidly and selectively affords the bay‐functionalized 1‐(iminophosphorane)‐12‐hydroxy PDI derivative **3b** in excellent yield (94%) (Scheme 1) [38]. The influence of additives was also investigated. The addition of triethylamine had no significant impact on either the reaction rate or the yield, whereas the presence of acetic acid slightly decreased the yield (80%) and significantly slowed the reaction. Finally, this visible‐light driven transformation was further extended to nitro‐PDI **1a**, providing derivative **3a** in 87% yield.

### 2.1 Mechanistic Investigation of the PDI‐NO_2_ to PDI‐NO Transformation

We first examined the possibility of a donor‐acceptor interaction between PDI‐NO_2_ **1b** and PPh_3_. The transformation of PDI‐NO_2_ **1b** into compound **3b** was therefore studied using a strictly stoichiometric amount of PPh_3_. Interestingly, although PDI‐NO_2_ is completely consumed, the isolated yield of compound **3b** drops to 30%–40%, indicating the occurrence of significant side reactions or unproductive pathways. This suggests that an excess of PPh_3_ plays a crucial role not only in promoting the desired transformation but also in suppressing competing photochemical processes, thereby enhancing selectivity and yield. Importantly, the addition of one equivalent of TEMPO, a well‐known radical scavenger [51], did not affect the outcome of the reaction, as PDI‐NO_2_ was still completely consumed and the isolated yield of compound **3b** remained in the same range (30%–40%). This observation further supports the hypothesis that, although a single‐electron transfer process is likely involved, the key reactive intermediates are either extremely short‐lived, generated within a “caged” environment [52], or react within a charge‐transfer complex, thereby preventing interception by TEMPO. Notably, no trace of Ph_3_P═O was detected by thin‐layer chromatography or infrared spectroscopy, as evidenced by the absence of the characteristic P═O band around 1150‐1200 cm^−^^1^ (Supporting Information, Figure S12). These observations strongly suggest that the initial deoxygenation of PDI‐NO_2_ follows a nonclassical pathway. This led us to hypothesize that PDI‐NO_2_ forms a charge‐transfer complex with PPh_3_ and behaves as a photoactive species, enabling a photocatalytic deoxygenation process that produces PDI‐NO with the release of molecular oxygen, rather than generating Ph_3_P═O. Considering the reduction potential of the radical anion at ‐0.83 V versus Fc^+^/Fc and the absorption maximum at *λ*_max_ = 519 nm (corresponding to *E*_0‐0_ = 2.39 eV) [38], the Rehm‐Weller equation indicates a very strong excited‐state reducing potential at ‐3.22 V versus Fc^+^/Fc. This value is fully consistent with the strong reducing power of the photoexcited PDI‐NO_2_ radical anion, which is sufficient to promote single‐electron transfer to the nitro group. Overall, these findings point toward an unconventional, PDI‐mediated photoredox mechanism that challenges the traditional stoichiometric role of phosphines in Cadogan‐type reactions.

### 2.2 Mechanistic Investigation of the Visible‐Light‐Driven Reaction Between PDI‐NO and PPh_3_

#### 2.2.1 Experimental Insights

**SCHEME 4:**
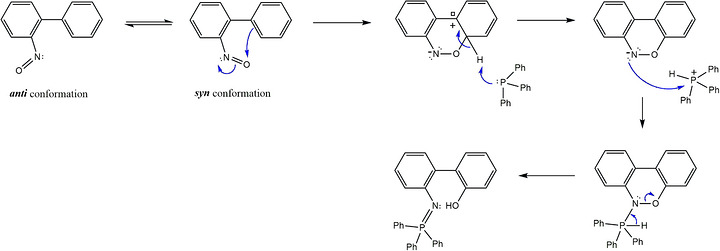
Proposed mechanism of the visible‐light driven competitive reaction to the Cadogan cyclization (compound 2‐nitrobiphenyl has been used as model to simplify the description of the proposed mechanism).

Our goal is to identify the factors that, upon reaction of PPh_3_ with the nitroso group, direct the process either toward carbazole formation or toward the competing photochemical pathway. To this end, a mechanistic proposal for the visible‐light‐driven pathway competing with the Cadogan cyclization is presented in Scheme 4 and further investigated theoretically. The study was performed starting from PDI‐NO, considering the full PDI framework to properly capture its specific electronic characteristics.

To gain deeper insight into this reaction and validate the proposed mechanism through theoretical calculations, an experimental protocol was designed to monitor reaction kinetics and evaluate the contribution of different spectral components of the LED irradiation. Three photochemical experiments were performed sequentially under identical conditions (Supporting Information, Figures S6‐S11). The reaction mixture, containing PDI‐NO_2_ **1** (0.10 mmol) and PPh_3_ (0.22 mmol) in 10 mL of anhydrous THF, was prepared under argon in a 25 mL round‐bottom flask. The flask was placed in a custom‐built light‐tight box and irradiated with the LED lamp while being stirred for 1 h to obtain partial conversion. After irradiation, the solvent was removed under reduced pressure, and the crude mixture was analyzed directly by ^1^H NMR spectroscopy. The relative proportions of PDI‐NO_2_ **1b** and compound **3b** were determined using two typical protons in close proximity on the spectrum: the doublet at 8.16 ppm for PDI‐NO_2_ **1b** and the singlet at 8.05 ppm for compound **3b**. Under full LED irradiation, compound **3b** was formed in 63% yield (Figure S9). To assess the wavelength dependence, the reaction was repeated using optical filters. Irradiation through a long‐pass filter (*λ* > 500 nm, blocking UV and blue light) yielded 24% of compound **3b** (Figure S10), whereas irradiation through a short‐pass filter (*λ* < 500 nm, blocking green and red light) yielded 36% (Figure S11). The spectral energy and photon flux distribution of the light source were calculated (Supporting Information), revealing that 26% of the photons were emitted in the 400‐500 nm range and 74% in the 500‐760 nm range. In conclusion, the formation of compound **3b** is strongly wavelength‐dependent and is predominantly driven by high‐energy blue photons (400‐500 nm). Although lower‐energy green and red photons (500‐760 nm) also contribute, their effect is significantly less pronounced despite their higher abundance. These results provide clear evidence of a wavelength‐dependent activation mechanism, highlighting the distinct roles of high‐ and low‐energy spectral components in the photochemical transformation of PDI‐NO_2_ **1b**. Complementary ^31^P NMR monitoring experiments were performed to follow the evolution of the phosphorus species during irradiation (Supporting Information, Figure S13). Time‐resolved spectra revealed the progressive consumption of PPh_3_ accompanied by the formation of triphenylphosphine oxide. No phosphorus‐containing intermediate was detected during the reaction, suggesting that any such species are short‐lived and present at concentrations below the detection limit of ^31^P NMR spectroscopy.

#### 2.2.2 Computational Details

To gain further insight into the reaction mechanism and the nature of the transient intermediates involved in this transformation, DFT and TD‐DFT calculations were performed using the Gaussian16 program [53]. To reduce computational costs, the R group of compound **3b** [R = ─C_6_H_11_] was substituted with a methyl group in all calculations. Geometry optimizations were carried out employing the PBE0 exchange correlation functional [54, 55] in conjunction with Pople 6‐311+G basis set, including diffuse functions for N, O, and P heteroatoms [56, 57]. The D3(BJ) dispersion correction was applied [58]. Implicit solvent effects (here tetrahydrofuran) were included using the integral equation formalism variant of the polarizable continuum model (IEFPCM, default in Gaussian16) [59, 60]. Analytical second derivatives of the energy were calculated to verify the nature of each stationary point, to determine the harmonic vibrational frequencies, and to provide zero‐point vibrational energy corrections. The thermal and entropic contributions to the free energies were also obtained using unscaled frequencies. Absorption spectra were simulated by Gaussian convolution of the first 25 vertical excitations, computed at TD‐DFT level and using a FWHM of 0.30 eV.

**FIGURE 1:**
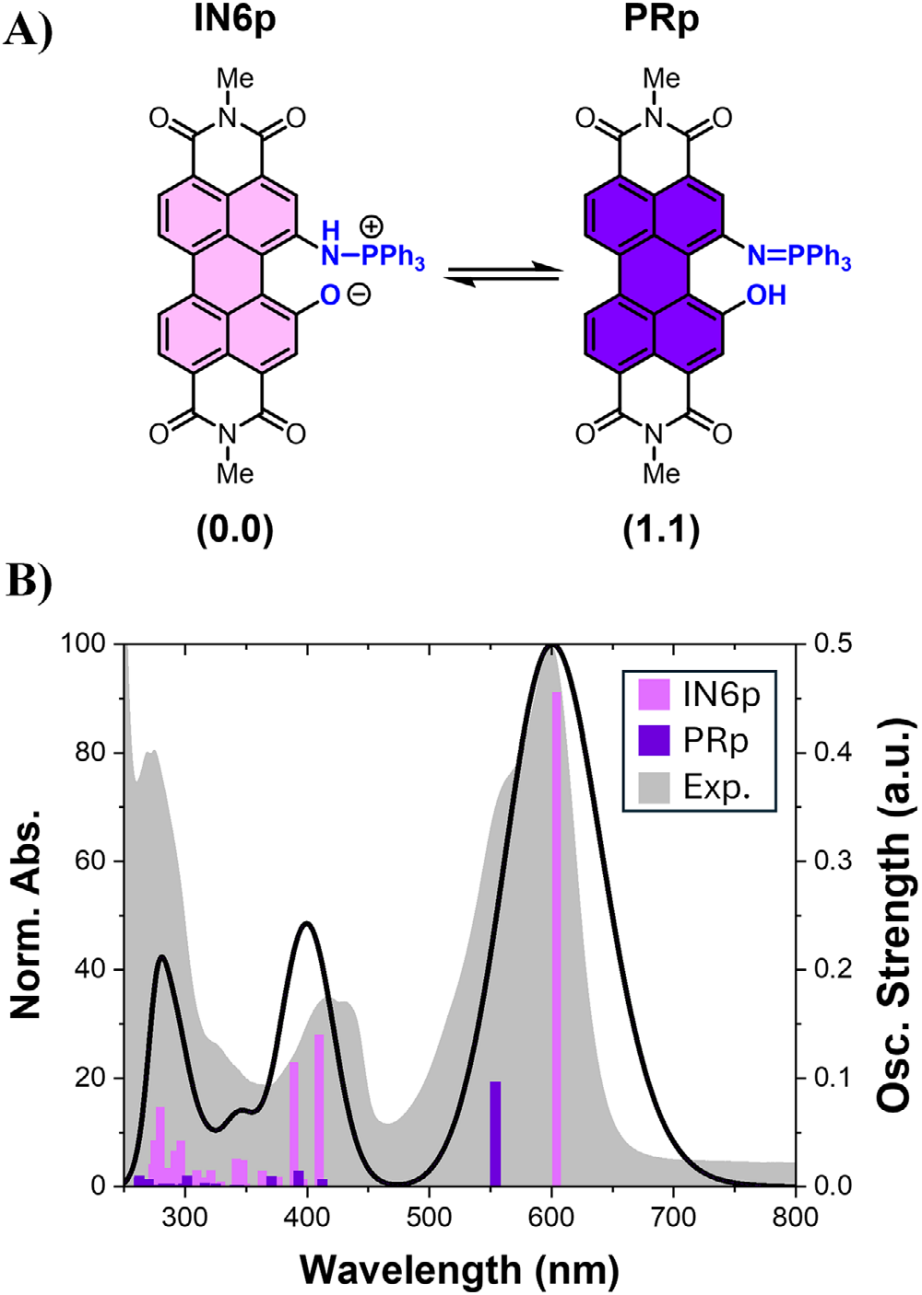
(A) Tautomeric equilibrium of the photoinduced addition product. Relative energies (in kcal·mol^−1^) are reported below each structure. (B) Comparison between the experimental spectrum (grey surface) and computed, convoluted spectrum (black trace). The spectral contribution of each tautomer is weighted according to their relative energy.

Since no clear consensus is found in literature on the methodology to be used to accurately describe the photophysical properties of PDI derivatives [61, 62, 63, 64], we firstly assessed the ability of the PBE0 exchange‐correlation functional together with the 6‐311+G basis set in the description of the absorption spectra of the product **PRp** (Figure 1), also experimentally available. Notably, this compound has been found to be in fast equilibrium with its prototropic tautomer **IN6p**. The computed convoluted spectrum, according to the Boltzmann distribution of both tautomers, and the experimental one are reported in Figure 1.

The good agreement between computed and experimental spectra confirms the suitability of the proposed computational approach and provides additional insight into the spectral features of the two prototropic tautomers (Supporting Information).

### 2.3 Thermally versus Light Activated Mechanism

In this section, we describe in detail the computed reaction pathways associated with the Cadogan reaction and with PPh_3_ addition, both on the ground state (GS) and on the excited‐state potential energy surfaces. In the latter case, the following labelling scheme is adopted to distinguish the different excited states: $nS_{\mathrm{type}}$where *type* represents the character of the excited state (here π−π∗, Charge Transfer (CT) or nitroso centred (NO)) and *n* refers to the excited state number for each type in increasing energy order (i.e., from the lowest lying ‐labelled 1‐ to the highest one).

**FIGURE 2:**
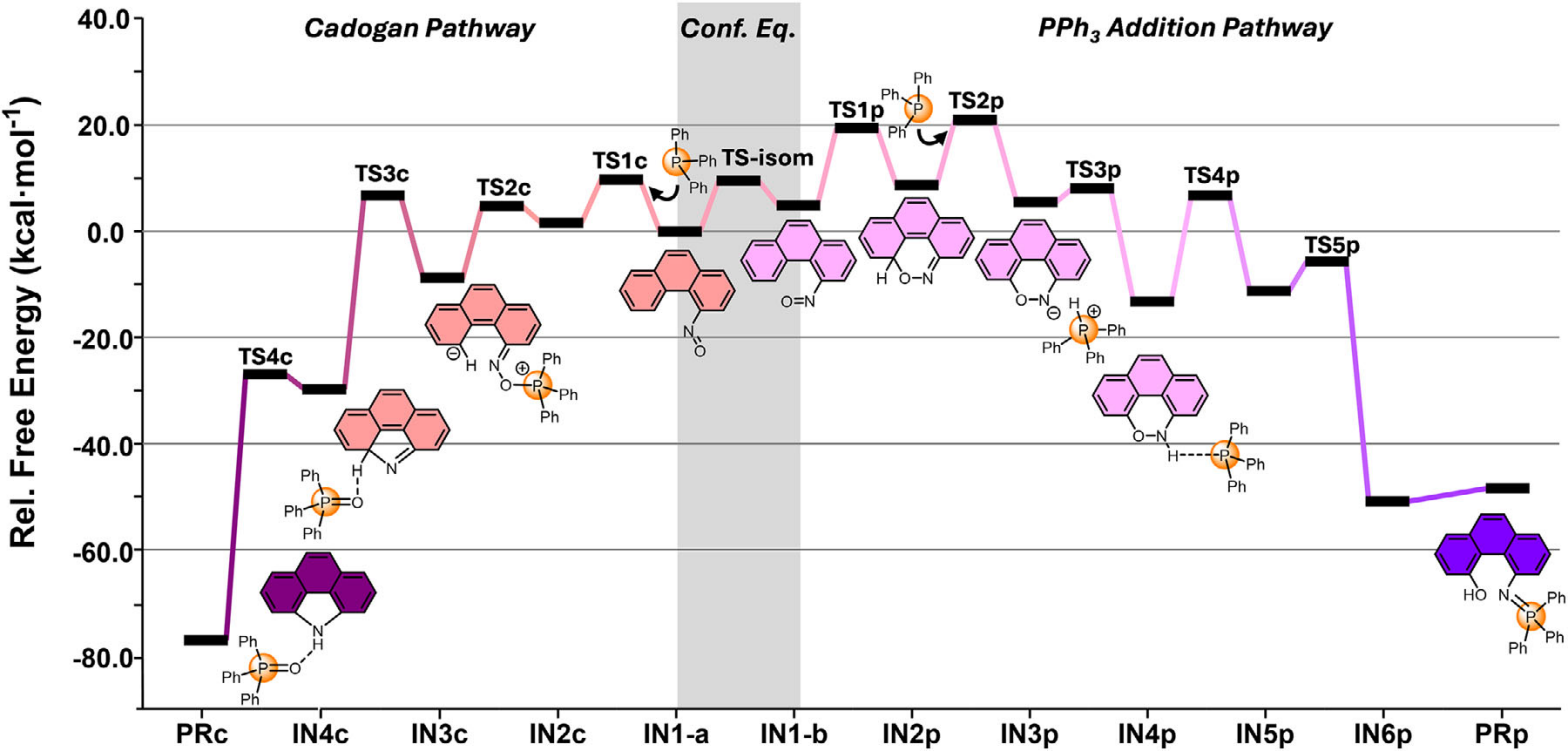
Relative free energy diagram for the competitive Cadogan and triphenylphosphine‐addition pathways from the conformational equilibrium (Conf. Eq.) of the nitroso intermediate. 4‐Nitrosophenanthrene has been used in the figure to simplify the PDI structure.

The Cadogan reaction being widely accepted to proceed through an initial nitroso intermediate [40], this species (**IN1**) was thus considered as the starting point for studying the reactivity of the PDI in presence and absence of irradiation. Computational results support the coexistence of two interconverting conformers, the *anti* (**IN1‐a**) and *syn* (**IN1‐b**) forms, which are in fast equilibrium. Each of these species plays the role of the starting structure for distinct reaction pathways. Notably, inclusion of the phosphine molecule at this stage did not alter the relative populations of the conformers nor the interconversion barrier (see Figures 2 and S14 and corresponding discussion in Supporting Information).

The Cadogan reaction pathway is initiated by a nucleophilic attack of PPh_3_ on the nitroso moiety of **IN1‐a**, with either N─ or O─attack leading to intermediates **IN2c** and **IN3c**, respectively. As described by M. Castiñeira Reis et al. for trimethylphosphine [], lower barriers were found for the attack at the nitrogen atom than at the oxygen (barriers of 9.4 and 16.9 kcal·mol^−1^, respectively), yielding intermediate **IN2c**, which then isomerises through a very small barrier (1.7 kcal·mol^−1^) to intermediate **IN3c**, thermodynamically more stable due to an increased delocalisation of electron density over the PDI skeleton. Contrary to previous report [65], no oxazaphosphiridine intermediate was found between **IN2c** and I**N3c**, likely due to the lower nucleophilicity of PPh_3_ compared with other aliphatic phosphines. From this point, all attempts to locate a stable nitrene intermediate were unsuccessful, in contrast with earlier literature [40, 65, 66]. Instead, oxidation of PPh_3_ was found to be concerted with the cyclisation toward the carbazole moiety, yielding **IN4c** with a barrier of 13.7 kcal·mol^−1^. This step is actually the rate‐determining step (RDS) of the Cadogan pathway. Finally, a barrierless rearomatisation, mediated by the newly formed Ph_3_P═O, produces the final product of the Cadogan pathway, **PRc** (Figure 2, left).

In contrast to the Cadogan reaction, the PPh_3_ addition pathway begins from the *syn‐*conformer **IN1‐b**, which undergoes an intramolecular pericyclic reaction to form **IN2p**, with a barrier of 19.2 kcal·mol^−1^. Although this intramolecular cyclisation has been reported on β‐nitroso‐*o*‐quinone methides [67], to the best of our knowledge this is the first time it has been described for PDI derivatives.

In the next step, PPh_3_ acts as a base, abstracting the hydrogen atom in the α‐position relative to the oxygen atom to form **IN3p**. This step is actually the RDS of the phosphine‐addition pathway and presents a barrier of 21.3 kcal·mol^−1^ at the GS. This finding is consistent with the predominant formation of the Cadogan reaction product under reflux conditions. Although the use of a stronger base (i.e., NEt_3_) is predicted to decrease this barrier (to 12.2 kcal·mol^−1^), this pathway must overcome the initial nitroso‐group cyclisation, which remains 5.5 kcal·mol^−1^ above the Cadogan RDS.

Next, the reprotonation of the **IN3p** anion was found to be more favourable than the formation of the hypervalent species depicted in Scheme 4, regenerating the phosphine molecule and yielding **IN4p**, the aromatic isomer of **IN2p**, which lies at 12.7 kcal·mol^−1^ below the starting nitroso intermediate. From this point, a nucleophilic attack of the phosphine molecule on the nitrogen atom allows the cleavage of the N─O bond, generating **IN6p** as the tautomer of the final product **PRp** in a concerted manner. However, this process was found to be 3.1 kcal·mol^−1^ higher in energy than the stepwise mechanism, in which cleavage of the N─O bond first leads to **IN5p**, followed by a rapid nucleophilic attack of the phosphine on the resulting primary imine. Finally, **IN6p** and **PRp** are predicted to be in fast equilibrium, their interconversion being barrierless. The complete competitive reactions pathway is depicted in Figure 2. A more detailed description, including the representation of all intermediates and all relative free energies, is presented in the Supporting Information (Figure S14).

Overall, under thermal activation in the GS, the Cadogan pathway is computed to be the most favourable in agreement with the experimental findings.

### 2.4 Light Activated Reactivity

**FIGURE 3:**
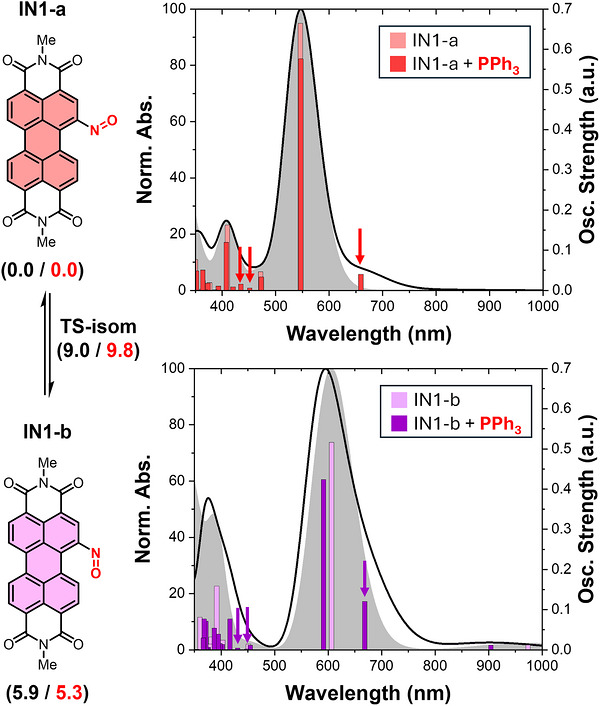
Computed absorption spectra for IN1‐a (top) and IN1‐b (bottom) in the absence (grey surface) and presence (black trace) of PPh_3_. Vertical bars indicate the oscillator strength of each transition. Relative energies with respect to IN1‐a of the conformers are shown in kcal·mol^−1^, with values in red corresponding to those computed in the presence of PPh_3_. Arrows indicate the lowest CT transitions.

To investigate the effect of light irradiation on reaction selectivity, TD‐DFT calculations were performed on both **IN1** conformers (**IN1‐a** and **IN1‐b**). Calculations were carried out in presence and absence of PPh_3_ to assess its impact on the photophysical properties. As shown in Figure 3, in absence of PPh_3_, the spectra of both conformers are characterized by two bands: an intense band with maximum at 550 and 600 nm, respectively for **IN1‐a** and **IN1‐b**, and a weaker band, around 400 nm. Both bands are associated to π‐π* transitions and characterized by a single dominant contribution. It is worth noting both conformers also show a very low‐lying nitroso‐centred transition, at 858 nm (**IN1‐a**) and 974 nm (**IN1‐b**), corresponding to a dark state.

After inclusion of a PPh_3_ molecule, several new excitations appear, all corresponding to charge‐transfer (CT) transitions from the phosphine to the PDI moiety. The lowest CT, observed around 660 nm for both conformers, corresponds to a transition from the HOMO of the phosphine, mainly corresponding to the phosphorous atom lone pair, to the π‐delocalised LUMO of the PDI ($1S_{\mathrm{CT}}$). A second CT transition from the phosphine to the PDI LUMO+1 is observed at 452 and 395 nm for **IN1‐a** and **IN1‐b**, respectively ($2S_{\mathrm{CT}}$). Although other transitions occur between these excitations, they are omitted in the discussion since they have a negligible impact on the photochemical behaviour of the system (see Supporting Information for further details).

**FIGURE 4:**
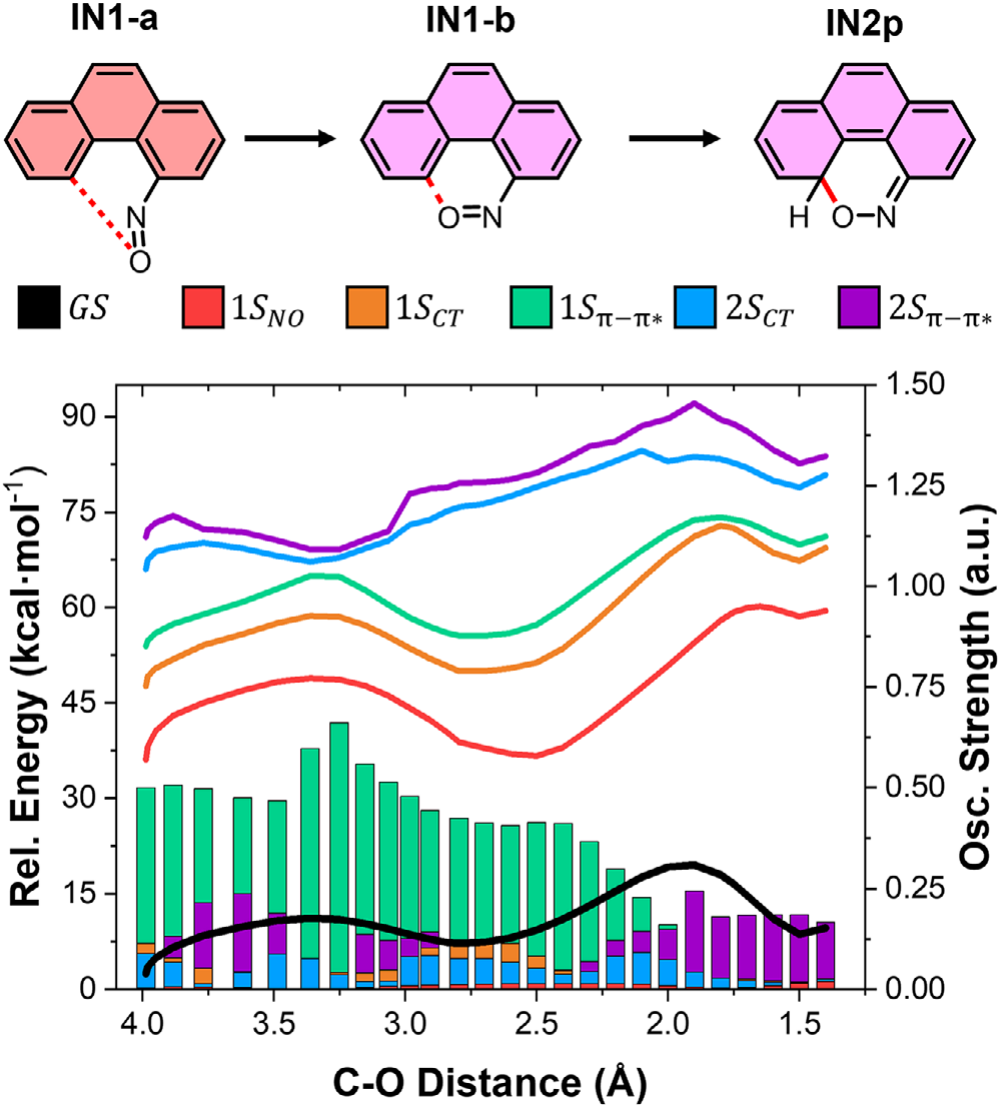
Vertical excitation energies along the relaxed C─O bond distance scan at the GS geometry, from IN1‐a to IN2p, with the absorbance of each excited state represented by oscillator strengths. Scanned distance has been marked in red, and 4‐nitrosophenanthrene has been used in the figure to simplify the PDI structure.

For the PPh_3_ addition pathway to outcompete the Cadogan reaction, both the first and second barriers (**TS1p** and **TS2p,** Figure 2) must be reduced under irradiation. Once **IN3p** is formed, **IN4p** can indeed be obtained almost barrierless. After this step the reaction becomes effectively irreversible, leading exclusively to the photoinduced product. To investigate the influence of light irradiation on these steps, relaxed scans along the C─O bond distance were performed from **IN1‐a** to **IN2p**, and vertical excitations were computed at each point. Natural Transition Orbitals (NTOs) were analysed to assess the nature of each excited state. Results for the lowest excited states are summarised in Figure 4.

Overall, the three lowest excited states exhibit behaviour distinct from the two higher states during the shortening of the C─O bond. Considering the conformational barrier between **IN1‐a** and **IN1‐b**, although this step remains feasible in the GS, the vertical excited state energies near this TS show a significant reduction in the energy gap between $1S_{\pi -\pi *}$, $2S_{\mathrm{CT}}$ and $\;2S_{\pi -\pi *}$ states, thereby facilitating nitroso group rotation thanks to internal conversion.

**FIGURE 5:**
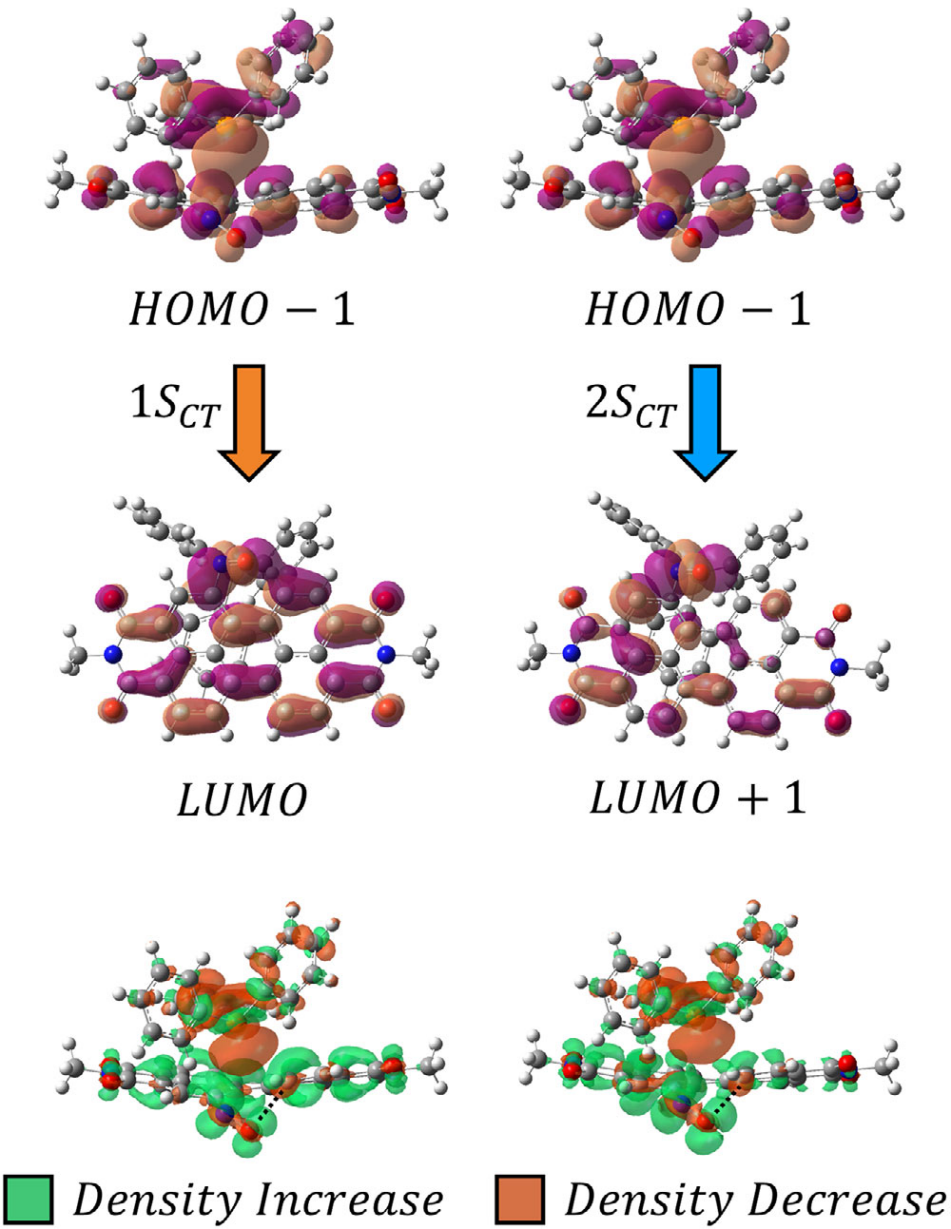
(Top) Isocontour representation of the MOs involved in the lowest CT states (isoval. = 0.02 a.u.); (Bottom) Difference between the electron density isocontours between the GS and the CT states (isoval. = 0.001 a.u.).

The second barrier (**TS2p**) is not competitive with the Cadogan pathway and therefore must be lowered in the excited state. Although a CT transition between the phosphine and the PDI moiety could, in principle, facilitate this process, none of the first three excited states, including the second, which has CT character, appears capable of reducing the barrier relative to the GS. Scans performed on the PDI radical anion support this conclusion, ruling out $1S_{CT}$ as responsible for the observed reactivity (Supporting Information). Comparison of the electron density between the GS and the first three excited states reveals no significant redistribution between the nitroso group and the electrophilic carbon of the PDI framework. Specifically, excitation does not lead to an increase in electron density at the nitroso group nor a decrease at the reactive carbon, which would have facilitated cyclization. Indeed, all these excitations involve population of the LUMO, which is symmetrically distributed across the PDI framework (Figure 5, top).

In contrast, the latter two excited states ($2S_{\mathrm{CT}}$ and $\;2S_{\pi -\pi *}$) populate the LUMO+1 orbital, which is localized on the nitroso group and adjacent aromatic rings rather than being symmetrically distributed. This results in increased electron density on the nitroso region while depleting electron density at the reactive carbon site (Figure 5, bottom), thereby facilitating the cyclization.

Excited state relaxation along the C─O bond distance reveals an almost barrierless cyclization at the $2S_{CT}$ excited state [68]. Considering the intrinsic error in the excited state energy evaluation using TD‐DFT, these excited states lie at the limit of what it can be efficiently excited below 500 nm, which may explain the lower yield obtained with the 500 nm longpass filter (Figure 3).

**FIGURE 6:**
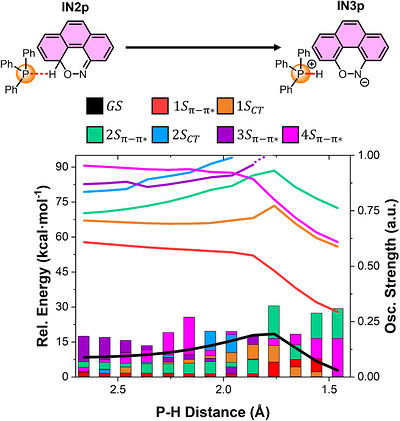
Vertical excitation energies along the GS‐relaxed P─H bond distance scan between IN2p and IN3p, with oscillator strengths shown as vertical bars for each excited state. Scanned distance has been marked in red, and 4‐nitrosophenanthrene has been used in the figure to simplify the PDI structure. The apparent TS on the $1S_{\pi -\pi *}$ surface is an artifact arising from state mixing with $3S_{\pi -\pi *}$. Excited state optimizations confirm a barrierless progression toward IN3p.

Although this explains how **IN2p** can be formed, the deprotonation of this intermediate through **TS2p** still requires overcoming a barrier of 12.2 kcal·mol^−1^, the highest TS in terms of free energy across the entire reaction mechanism and 21.3 kcal·mol^−1^ above the starting intermediate **IN1‐a**. Moreover, as shown in Figure 6, the energy gap between the GS and the excited states of **IN2p** increases relative to both **IN1** conformers, reducing the number of states accessible under the current irradiation conditions to only three.

Optimization of the first two excited states of **IN2p** (now the first one being of a π‐π* character due to the change in electronic structure induced by the pericyclic reaction) resulted in spontaneous deprotonation by the phosphine and concomitant planarization of the PDI framework. Motivated by this behaviour, an additional vertical excitation analysis was carried out along the GS‐relaxed scan of the P─H bond distance. This scan revealed a barrierless photoinduced H‐transfer occurring in the two lowest excited states of the intermediate ($1S_{\pi -\pi *}$ and $1S_{\mathrm{CT}})$, both of which are accessible at the wavelengths investigated (Figure 6).

Comparison of the electron density between the GS and each corresponding excited state revealed that for the first two excited states, the migrating hydrogen atom experiences a relative increase in electron density at the transition state, accompanied by a decrease at both the carbon and phosphorus atoms. This redistribution accounts for the observed barrierless behaviour on these surfaces. In contrast, such density variations are negligible for $1S_{\mathrm{CT}}$, consistent with its inability to promote the H‐transfer process (Supporting Information).

## 3 Conclusion

In conclusion, the reactivity of the bay‐nitrated perylenediimide (PDI‐NO_2_) derivative toward triphenylphosphine has been elucidated under both thermal and photochemical conditions, revealing two fundamentally distinct mechanistic pathways. Under thermal activation, the reaction proceeds preferentially through the known Cadogan‐type transformation. While the overall transformation remains well‐established, mechanistic analysis reveals a notable deviation from the classical mechanism, in which the nitrene intermediate is replaced by a concerted phosphine oxidation‐carbazole cyclization step that constitutes the rate‐determining process. In contrast, direct phosphine addition to the nitroso intermediate is intrinsically disfavored in the GS due to the presence of two high‐energy barriers associated with nitroso‐group cyclization and subsequent hydrogen abstraction, effectively preventing any competition with the Cadogan route.

However, visible‐light irradiation selectively redirects the reaction toward a previously unobserved pathway, yielding a bay‐functionalized 1‐(iminophosphorane)‐12‐hydroxy PDI derivative in excellent yield. Initial experimental investigations reveal that the conversion of PDI‐NO_2_ into the corresponding nitroso intermediate (PDI‐NO) does not require phosphine‐mediated deoxygenation, but instead more probably arises from the intrinsic reducing power of the photoexcited PDI‐NO_2_. Systematic analysis of the spectral components of white LED irradiation demonstrates a strong wavelength dependence, with high‐energy blue photons (400‐500 nm) playing a dominant role in driving the photochemical transformation, while lower‐energy green and red photons contribute only marginally. TD‐DFT calculations demonstrate that selective population of excited states involving the LUMO+1, an asymmetric orbital localized on the nitroso moiety and adjacent aromatic framework, induces a pronounced electronic imbalance that unlocks a cyclization pathway inaccessible under thermal conditions.

Furthermore, the second key barrier, corresponding to hydrogen abstraction, is overcome through a barrierless photoinduced hydrogen‐atom transfer from intermediate **IN2p** occurring on low‐lying excited states characterized by increased electron density on the abstracted hydrogen atom. The selective population of these states, depending on the irradiation wavelength, provides a direct mechanistic rationale for the higher efficiency observed under shorter‐wavelength excitation.

Overall, this study demonstrates that photoexcitation does not merely accelerate a ground‐state reaction pathway but instead enables access to an entirely distinct mechanistic channel governed by orbital‐specific excitation and excited‐state hydrogen transfer. These findings establish a general conceptual framework for understanding and exploiting light‐gated chemoselectivity in nitroso‐aromatic systems and PDI‐based π‐conjugated scaffolds.

## Conflicts of Interest

There are no conflicts to declare.

## Supporting information




**Supporting File**: chem70970‐sup‐0001‐SuppMat.docx

## Data Availability

The data that supports the findings of this study are available in the supplementary material of this article.
