# Supplementary material for: When Light Challenges Heat: Mechanistic Insights Into a Reaction Competing With Cadogan Cyclisation in Nitro‐Perylenediimides
Source: Chemistry. 2026 Apr 9;32(24):e70970. doi: 10.1002/chem.70970 (PMC13290422; doi:10.1002/chem.70970)
Supplement: Supplementary file 1 — Supporting File: chem70970‐sup‐0001‐SuppMat.docx [file CHEM-32-e70970-s001.docx]

**SUPPOR**TIN**G INFORMATION**

**When Light Challenges Heat: Mechanistic Insights into a Reaction Competing with Cadogan cyclisation in Nitro-Perylenediimides**

Manuel Pedrón Laserna,^1,2^ Ilaria Ciofini,^1,*^ Piétrick Hudhomme^3,*^

^1^ Chimie Paris Tech, PSL University, CNRS, Institute of Chemistry for Life and Health Sciences, 75005 Paris, France

^2^ Institute of Biocomputation and Physics of Complex Systems (BIFI), University of Zaragoza, 50009 Zaragoza, Spain

^3^ Univ Angers, CNRS, MOLTECH-Anjou, SFR MATRIX, F- 49000 Angers, France.

E-mail: [ilaria.ciofini@chimieparistech.psl.eu](mailto:ilaria.ciofini@chimieparistech.psl.eu); [pietrick.hudhomme@univ-angers.fr](mailto:pietrick.hudhomme@univ-angers.fr)

Table of content

[I. Materials and Methods 3](#_Toc224724265)

[II. Experimental Procedures 4](#_Toc224724266)

[III. Kinetics studies 8](#_Toc224724267)

[IV. Theoretical Calculations 15](#_Toc224724268)

[IV.1 Relative energies 15](#_Toc224724269)

[IV.2 PDI anion radical calculations 19](#_Toc224724270)

[IV.3 TD-DFT absorption spectra 19](#_Toc224724271)

[IV.4 Vertical excitations along scan coordinates 29](#_Toc224724272)

[IV.4.1 IN1-a scan to IN1-b 30](#_Toc224724273)

[IV.4.2 IN1-b scan to IN2p 32](#_Toc224724274)

[IV.4.3 IN2p scan to IN3p 35](#_Toc224724275)

[IV.5 Excited state optimizations along scan coordinates 37](#_Toc224724276)

[IV.6 Molecular Orbitals 38](#_Toc224724277)

[IV.7 Electronic density difference across excited states 42](#_Toc224724278)

## Materials and Methods

**Chemicals** were purchased from Alfa Aesar (triphenylphosphine, TEMPO). **Solvents** were purchased from Fisher Scientific (THF HPLC grade), deuterated solvent (CDCl_3_) from Sigma Aldrich. THF was dried over Na/benzophenone.

PDI starting materials **1a^[[1]](#footnote-1)^** and **1b**^[[2]](#footnote-2)^ were prepared according to literature.

^1^H, ^13^C, and ^31^P nuclear magnetic resonance (NMR) spectra were recorded on a Bruker Avance III HD 500 MHz spectrometer operating at 500 MHz for ^1^H, 125 MHz for ^13^C, and 202.4 MHz for ^31^P. ^13^C and ^31^P spectra were acquired with complete proton decoupling. Chemical shifts (δ) are reported in ppm relative to the residual solvent signal used as an internal reference (CDCl_3_: δ_H_ = 7.26 ppm, δ_C_ = 77.16 ppm). Coupling constants (J) are given in Hz. Signal multiplicities are reported as s (singlet), t (triplet), m (multiplet), and br s (broad singlet).

Thin layer chromatography (TLC) was conducted on pre-coated aluminum sheets with 0.20 mm Merck Alugram SIL G/UV254 with fluorescent indicator UV254. Column chromatography was carried out using Sigma-Aldrich silica gel 60 (particle size 63-200 μm).

The irradiation was done with a HCK1012-01-005 EvoluChem LED Spotlight (18 W) from HepatoChem (Figure S1). Some reactions were carried out in the presence of the filter FEL0500 Longpass Filter, Cut-On Wavelength: 500 nm or the filter FESH0500 Shortpass Filter, Cut-Off Wavelength: 500 nm, both filters purchased from Thorlabs


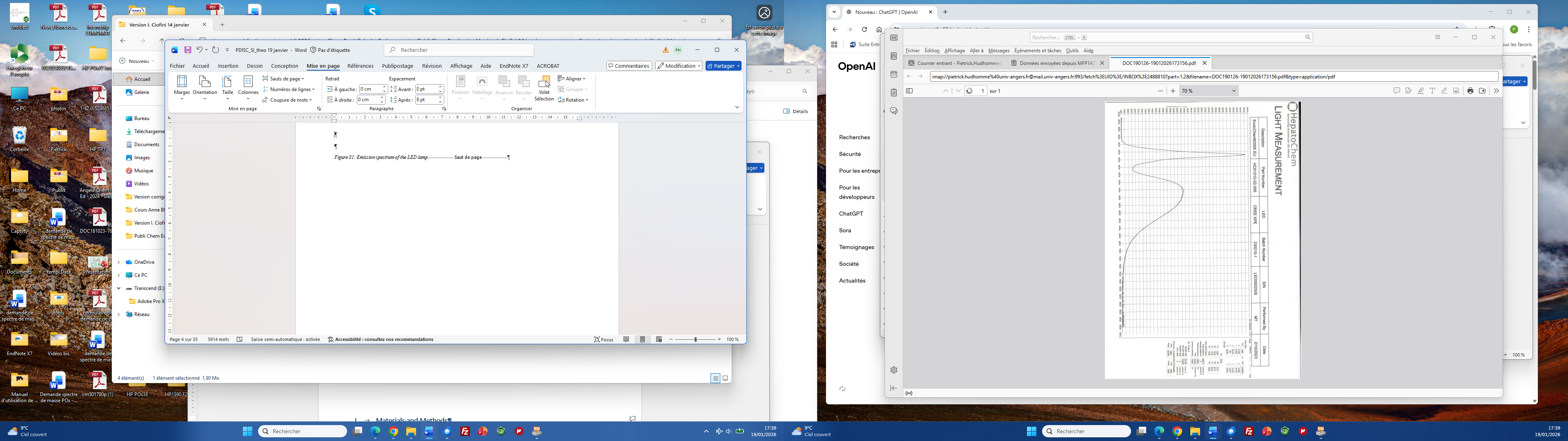


**Figure S1:** Emission spectrum of the LED lamp.

## Experimental Procedures

Compound **3b** was previously described in our earlier work, including its experimental synthetic procedure, spectroscopic characterizations, optical and electrochemical properties, and thermogravimetric analysis.^[[3]](#footnote-3)^

Compound **3a**: A solution of PDI-NO_2_ (575 mg, 1 mmol) and PPh_3_ (865 mg, 3.3 mmol) in anhydrous THF (100 mL) in a round-bottom flask was irradiated under white LED lamp for two hours. The solution was concentrated then the crude material was purified by silica gel chromatography using CH_2_Cl_2_ then CH_2_Cl_2_/EtOAc (95/5) as the mixture of eluents. After precipitation in methanol, a dark-violet powder was obtained (714 mg, yield: 87%).

^1^H NMR (500 MHz, CDCl_3_, 293 K, δ): 12.78 (br.s, 1H), 8.60-8.51 (m, 4H), 8.44 (s, 1H), 8.06 (s, 1H), 7.75 – 7.71 (m, 6H), 7.63 – 7.60 (m, 3H), 7.53 – 7.49 (m, 6H), 5.15-5.08 (m, 1H), 4.94 (m, 1H), 2.34-2.28 (m, 2H), 2.21-2.12 (m, 2H), 1.99-1.91 (m, 2H), 1.88-1.83 (m, 2H), 0.95 (t, J = 7Hz, 6H), 0.83 (t, J = 7 Hz, 6H).

^13^C NMR (125 MHz, CDCl_3_, 293 K, δ): 158.53, 144.09, 144.06, 133.77, 133.59, 133.57, 133.17, 133.04, 132.96, 129.72, 129.63, 129.53, 129.43, 128.01, 127.87, 126.72, 125.92, 124.44, 124.26, 122.06, 121.87, 121.84, 57.51, 57.30, 25.27, 25.07, 11.54, 11.40.

^31^P NMR (202.4 MHz, CDCl_3_, 293 K, δ): 20.85.

HR-MS (MALDI, DCTB, positive mode) m/z [M]^●+^ calc for [C_52_H_44_N_3_O_5_P]^●+^: 821.3013; found 821.3013 (0.03 ppm error).

**Figure S2:** ^1^H NMR (500 MHz, CDCl_3_) of compound **3a,** enlargements of the aromatic and aliphatic regions.

**Figure S3:** ^13^C NMR (125 MHz, CDCl_3_) of compound **3a**.

**Figure S4:** ^31^P NMR (202.4 MHz, CDCl_3_) of compound **3a**.


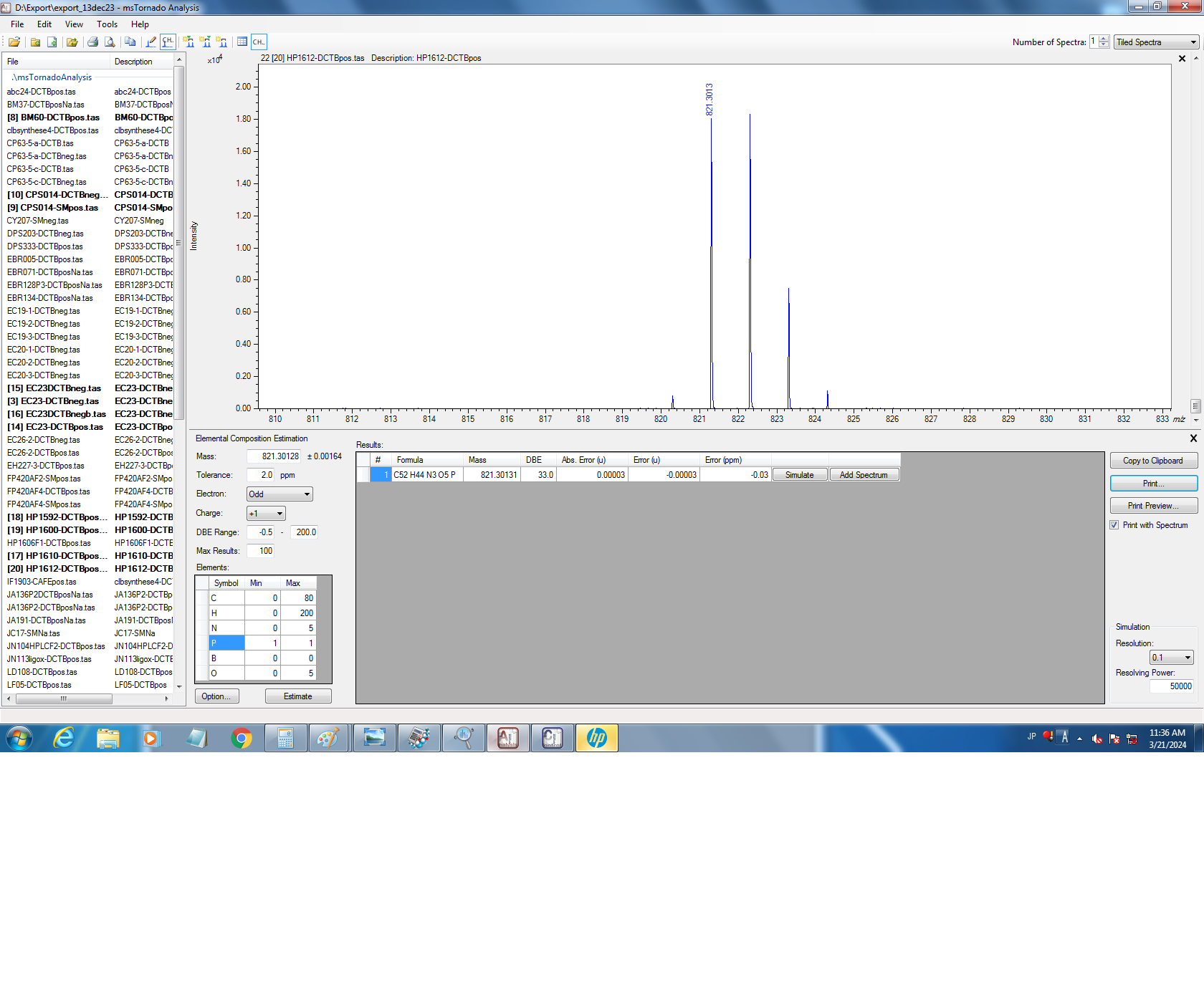


**Figure S5:** HR-MS (MALDI-TOF, DCTB matrix) of compound **3a**.

## Kinetics studies

Photochemical experiments were performed successively in the same 25 mL round-bottom flask equipped with the same magnetic stir bar, in order to ensure identical conditions for all three runs. The flask was placed inside a custom-made light-tight box, shielding it from any external light. The only illumination came from the LED lamp (see photograph below). The distance between the light source and the flask was carefully controlled and kept identical for all experiments.

In a 25 mL flask, PDI–NO_2_ **1b** (60 mg, 0.10 mmol) and PPh_3_ (58 mg, 0.22 mmol) were dissolved in 10 mL of anhydrous THF under an argon atmosphere. The mixture was stirred at 1400 rpm and irradiated for exactly 1 h. After the reaction, THF was evaporated under reduced pressure, and the crude product was analyzed directly by ^1^H NMR spectroscopy.


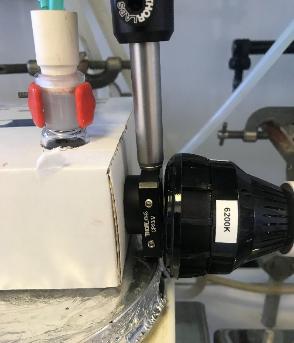

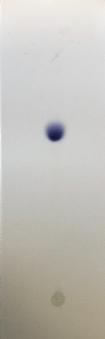


**Figure S6:** Photograph of the home-made experimental setup used to study the photochemical reaction and the effect of the light flux (left). Thin-layer chromatography after complete reaction (CH_2_Cl_2_/EtOAc 98:2 as eluent)

PPh_3_

**Figure S7:** ^1^H NMR spectra of compound **3** (top), PPh_3_ (middle), and nitroPDI **1b** (bottom)

*H_2_*

*H_2_*

*H_11_*

1H

PPh_3_

*H_12_*

1H

**Figure S8:** Enlarged view highlighting the selected characteristic protons

**I. Reaction of nitroPDI in the presence of PPh_3_:** **Experiment with total irradiance of LED lamp**


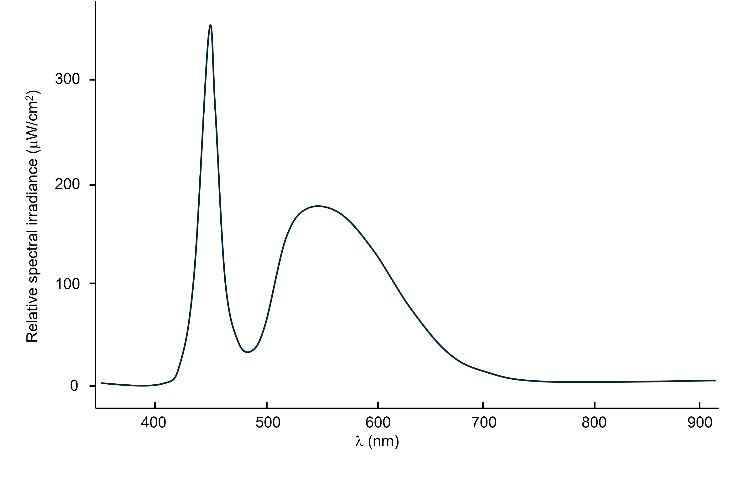

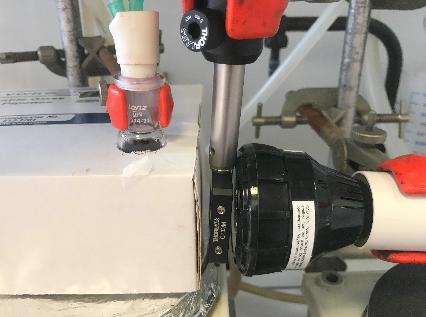

***

***

**Figure S9:** Photoirradiation of nitro-PDI **1b** with PPh_3_ under full-spectrum LED irradiation

**II. Reaction of nitroPDI in the presence of PPh_3_:** **Experiment in the presence of filter 500 Longpass Filter, Cut-On Wavelength: 500 nm**


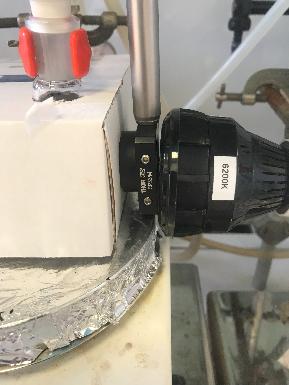


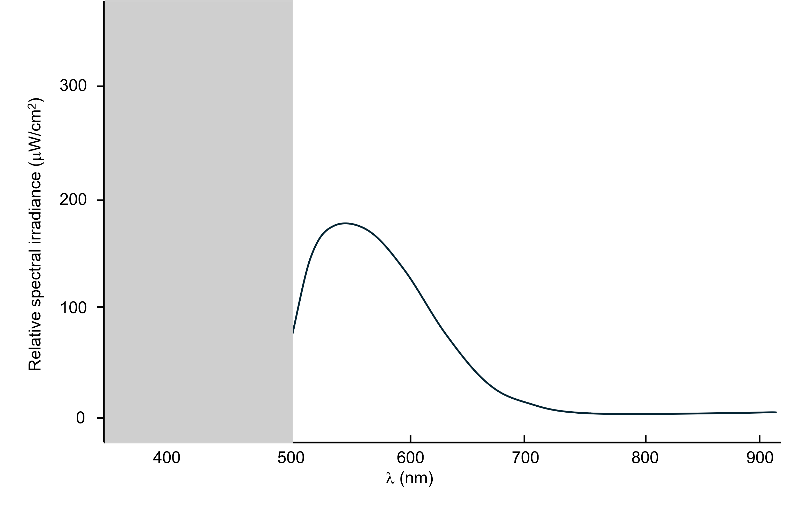


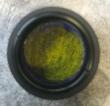

***

***

**Figure S10:** Photoirradiation of nitro-PDI **1b** with PPh_3_ under LED illumination using a 500 longpass filter (cut-on wavelength: 500 nm)

**III. Reaction of nitroPDI in the presence of PPh_3_:** **Experiment in the presence of filter 500 Shortpass Filter, Cut-Off Wavelength: 500 nm**


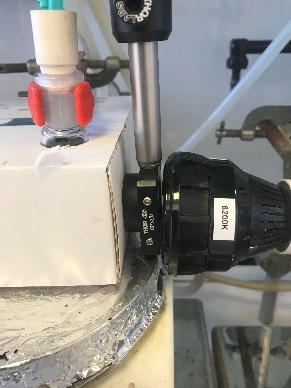

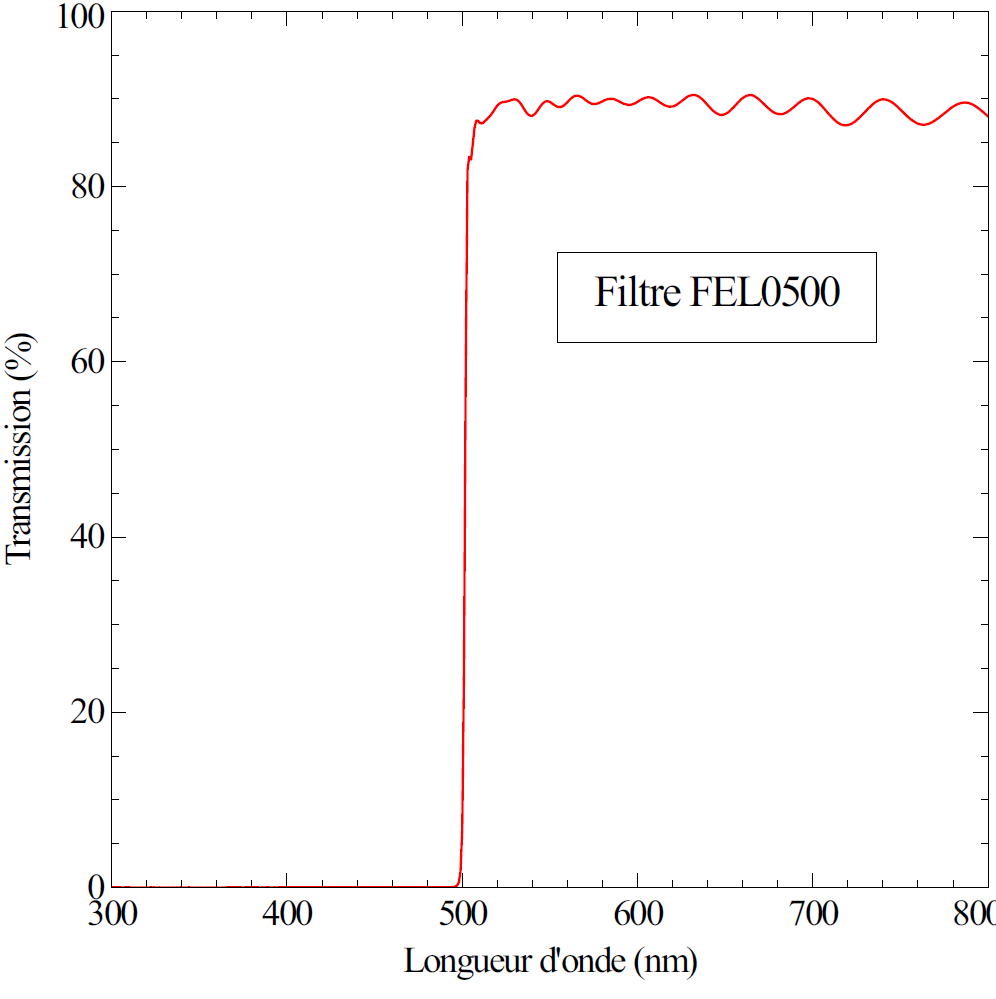


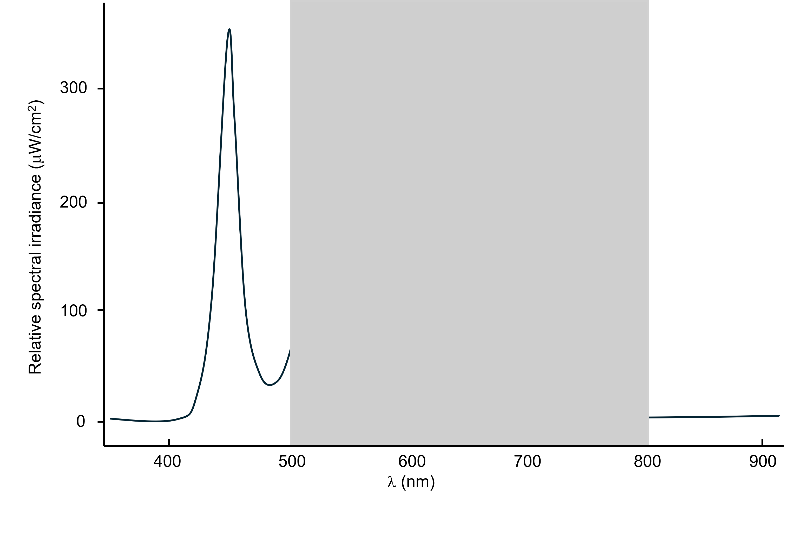


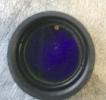

***

***

**Figure S11:** Photoirradiation of nitro-PDI **1b** with PPh_3_ under LED illumination using a 500 longpass filter (cut-off wavelength: 500 nm)

The spectral energy and the photon flux distribution of the light source were determined using the curve-weighing method. The printed emission spectrum was cut and weighed in two wavelength domains (400-500 nm and 500-760 nm). The relative paper weights were converted into energy fractions and then into photon fractions, taking representative wavelengths of 450 nm and 550 nm, respectively. The total luminous intensity of the lamp was 102 331 cd, assumed isotropic. The corresponding luminous flux (1.29 x 10^6^ lm) was converted to radiant power using an average luminous efficacy of 683 lm·W⁻¹. The resulting total optical power was 1.88 x 10^3^ W, leading to a total photon flux of 8.18 x 10⁻^3^ mol photons·m⁻^2^.s⁻^1^, with 26 % of photons in the 400-500 nm range and 74 % in the 500-760 nm range.

| **Wavelength range (nm)** | **Energy fraction (%)** | **Photon fraction (%)** | **Optical power (W)** | **Photons·s⁻^1^** | **Photon flux (mol·m⁻^2^·s⁻^1^)** |
| --- | --- | --- | --- | --- | --- |
| 400-500 | 30.00 | 25.96 | 564.85 | 1.28 x 10^21^ | 2.12 x 10⁻**^3^** |
| 500-760 | 70.00 | 74.04 | 1 317.92 | 3.65 x 10^21^ | 6.06 x 10⁻**^3^** |
| **Total** | **100** | **100** | **1 882.77** | **4.93 x 10^21^** | **8.18 x 10⁻^3^** |

Rehm-Weller equation :

$$E_{\text{red}}^{*}(\text{PDI•⁻})=E_{\text{red}}(\text{PDI•⁻})-E_{0-0}\approx-0.83-2.39=-3.22\text{ V vs }\text{Fc}\text{⁺/}\text{Fc}$$
***Figure S12:*** *Infrared spectra of pure compound* ***3****, and of crude reaction mixtures without and with TEMPO, for comparison with Ph₃P=O.*

**IV. ^31^P NMR monitoring of the photochemical reaction of PDI-NO₂ with triphenylphosphine**

In a 25 mL flask, PDI–NO₂ **1b** (60 mg, 0.10 mmol) and PPh₃ (58 mg, 0.22 mmol) were dissolved in 10 mL of anhydrous THF under an argon atmosphere. The reaction mixture was irradiated using an LED lamp. Aliquots (100 µL) were withdrawn at different time intervals and diluted with 0.5 mL of CDCl₃ prior to analysis. ^31^P NMR monitoring revealed the progressive consumption of PPh_3_ accompanied by the formation of triphenylphosphine oxide and compound **3b**. No phosphorus-containing intermediate could be detected during the course of the reaction.


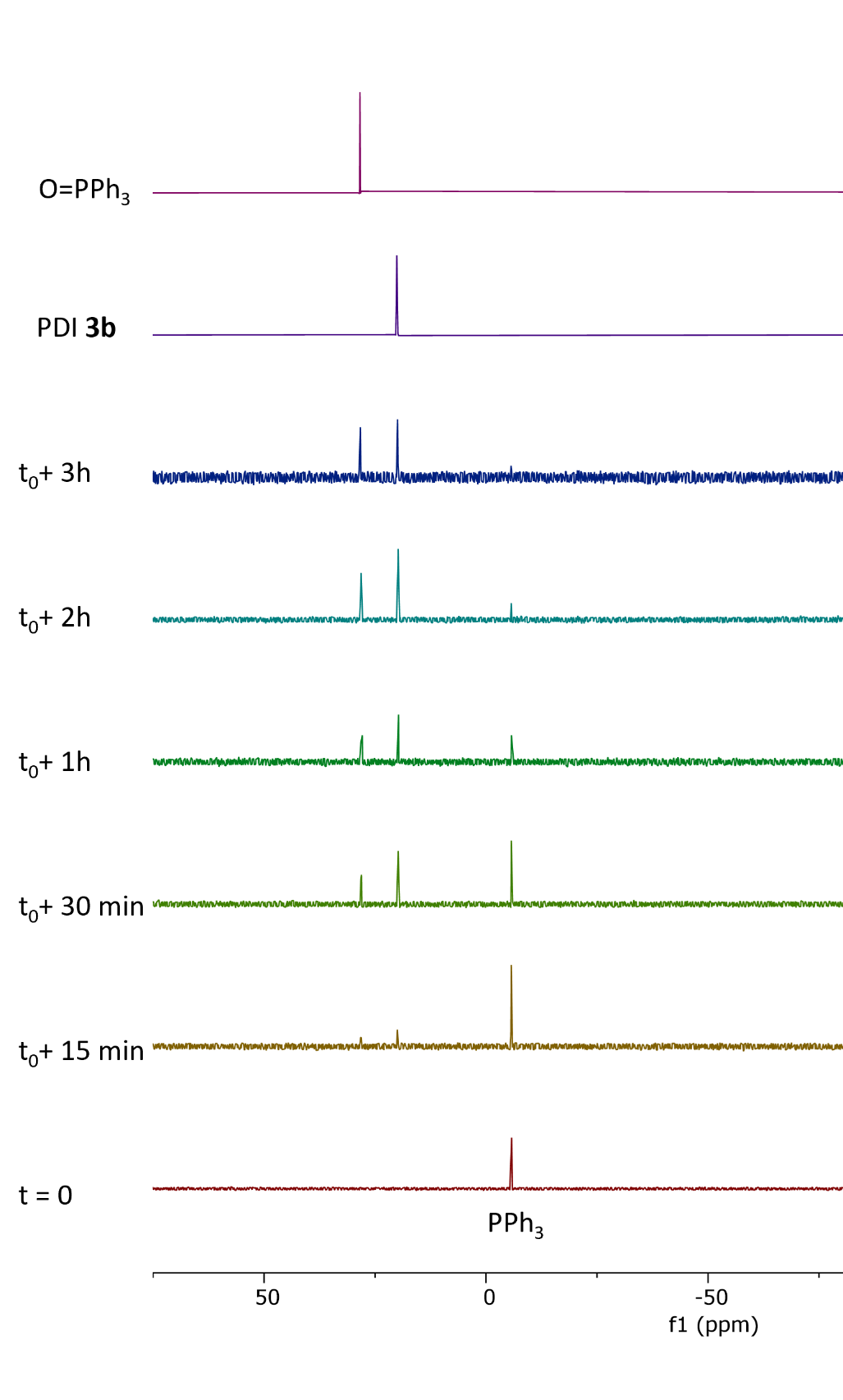


***Figure S13:*** *Time-dependent ^31^P NMR spectra monitoring the photochemical reaction of PDI–NO_2_* ***1b*** *with PPh_3_ in THF under LED irradiation.*

## Theoretical Calculations

### IV.1 Relative energies

**Table S1:** Calculated relative energies (kcal·mol^-1^) and first vibrational frequencies for the isolated PDI system.

| **Name** | **ΔE** | **ΔE_0_** | **ΔG** | **Freq.** |
| --- | --- | --- | --- | --- |
| IN1-a | **0.0** | **0.0** | **0.0** | 29.3 |
| TS-isom | **9.5** | **8.7** | **9.0** | -123.4 |
| IN1-b | **6.7** | **6.3** | **5.9** | 30.0 |
| TS1p | **17.7** | **17.8** | **19.2** | -383.3 |
| IN2p | **6.7** | **8.0** | **9.1** | 31.7 |

All relative energies for the compounds involved in the Cadogan reaction mechanism are collected in **Table S2**. Data for unfavourable pathways, such as the attack of the phosphine to the oxygen atom from the nitroso group (**TS1c-PO**) are also provided.

**Table S2:** Calculated relative energies (kcal·mol^-1^) and first vibrational frequencies for the Cadogan reaction pathway.

| **Name** | **ΔE** | **ΔE_0_** | **ΔG** | **Freq.** |
| --- | --- | --- | --- | --- |
| IN1-a-PPh_3_ | **0.0** | **0.0** | **0.0** | 8.3 |
| TS1c | **5.4** | **6.0** | **9.4** | -92.6 |
| TS1c-PO | **14.7** | **14.8** | **16.9** | -228.8 |
| IN2c | **-2.8** | **-1.5** | **2.2** | 15.9 |
| TS2c | **-0.7** | **0.4** | **3.9** | -152.2 |
| IN3c | **-9.2** | **-8.8** | **-7.9** | 9.3 |
| TS3c | **3.6** | **3.3** | **5.8** | -190.7 |
| IN4c | **-31.9** | **-31.0** | **-28.9** | 12.8 |
| TS4c | **-31.8** | **-31.9** | **-28.4** | -68.3 |
| PRc | **-81.2** | **-78.6** | **-76.1** | 10.7 |

**Table S3** compiles all relative energies and first vibrational frequencies for the ground-state triphenylphosphine addition pathway. Discarded pathways, such as the concerted N-O bond cleavage coupled with the H-transfer to the phosphine in **IN4p** (**TS4p-N**), or the deprotonation of **IN2p** with triethylamine are also included.

**Table S3:** Calculated relative energies (kcal·mol^-1^) and first vibrational frequencies for the ground-state PPh_3_ addition pathway.

| **Name** | **ΔE** | **ΔE_0_** | **ΔG** | **Freq.** |
| --- | --- | --- | --- | --- |
| IN1-a-PPh_3_ | **0.0** | **0.0** | **0.0** | 8.3 |
| TS-isom-PPh_3_ | **9.4** | **8.8** | **9.8** | -125.5 |
| IN1-b-PPh_3_ | **5.7** | **5.5** | **5.3** | 4.8 |
| TS1p-PPh_3_ | **16.2** | **16.4** | **18.1** | -367.5 |
| IN2p-PPh_3_ | **6.7** | **8.2** | **9.5** | 5.2 |
| TS2p | **20.8** | **18.6** | **21.3** | -1228.5 |
| IN3p | **2.4** | **2.3** | **5.1** | 6.7 |
| TS3p | **5.8** | **3.8** | **6.8** | -637.1 |
| IN4p | **-17.0** | **-15.2** | **-12.7** | 14.8 |
| TS4p | **4.2** | **4.4** | **6.4** | -587.5 |
| TS4p-N | **4.9** | **5.9** | **9.5** | -523.1 |
| IN5p | **-14.1** | **-13.0** | **-11.3** | 14.8 |
| TS5p | **-11.7** | **-10.4** | **-6.4** | -109.8 |
| IN6p | **-56.4** | **-54.4** | **-50.0** | 17.8 |
| TS6p | **-54.2** | **-54.7** | **-50.4** | -1005.4 |
| PRp | **-55.2** | **-53.3** | **-48.9** | 19.5 |
| IN2p-NEt_3_ | **0.0** | **0.0** | **0.0** | 11.0 |
| TS2p-NEt_3_ | **4.0** | **1.4** | **3.1** | -954.5 |

Finally, the relative energies of the TD-DFT optimized excited states for the first reaction intermediates are collected in **Table S4**.

**Table S4:** Calculated relative energies (kcal·mol^-1^) and first vibrational frequencies for the optimized excited states of **IN1-a**, **IN1-b**, and **IN2p**.

| **Name** | **ΔE** | **ΔE_0_** | **ΔG** | **Freq.** |
| --- | --- | --- | --- | --- |
| IN1-a-PPh_3_ | **0.0** | **0.0** | **0.0** | 8.3 |
| IN1-a-S1CT | **21.5** | **22.3** | **24.4** | 12.7 |
| IN1-a-S1pipi | **47.0** | **45.5** | **45.9** | 10.4 |
| IN1-a-S2CT | **44.5** | **44.6** | **47.0** | 13.1 |
| IN1-b-PPh_3_ | **5.7** | **5.5** | **5.3** | 4.8 |
| IN1-b-S1CT | **26.5** | **27.9** | **30.1** | 8.3 |
| IN1-b-S1pipi | **48.5** | **47.6** | **48.8** | 5.4 |
| IN2p-PPh_3_ | **6.7** | **8.2** | **9.5** | 5.2 |
| IN2p-S1CT^a^ | **18.5** | **16.8** | **19.4** | 13.3 |
| IN2p-S1pipi^b^ | **60.2** | **60.3** | **61.8** | 10.8 |
| IN2p-S2CT | **53.6** | **54.7** | **57.3** | 10.4 |

^a^ Barrierless optimization towards IN3p excited state. ^b^ Distinct starting geometries ended up in the barrierless optimization towards IN3p.

The complete energy profile of the two competing ground-state reactions is depicted in **Figure S14**.

**Figure S14:** Free energy profile (in kcal·mol^-1^) of the Cadogan (left) and PPh_3_ addition (right) competitive reaction pathways.

### IV.2 PDI anion radical calculations

To initially assess the feasibility of a charge transfer (CT) mechanism in explaining the observed photochemical reactivity, relaxed scans along the C-O bond distance were performed for both the neutral and radical-anionic states of the PDI molecule to evaluate the effect of the extra electron on the first two TSs. The results, shown in **Figure S15**, indicate an increase in the energy barriers for both **TS-isom** and **TS1p** relative to the ground state. Although these results argue against CT as a plausible explanation, as discussed in the main text, it is not the population of the LUMO, but rather the LUMO+1 that is responsible for lowering the barrier. The observed barrier increase is therefore fully consistent with further experiments, since in these calculations the extra electron occupies the LUMO.

**Figure S15:** Relaxed scan over the C-O bond distance between **IN1-a** and **IN2p** at both neutral and radical-anion states. Results show an increase in both barriers in the radical-anionic state.

### IV.3 TD-DFT absorption spectra

Results for the first 25 vertical excitations of both **IN1** conformers, in the presence and absence of the triphenylphosphine molecule, as well as those of **IN2p**, **IN3p**, and the two prototropic isomers of the final product (**IN6p** and **PRp**), are collected in **Tables** **S5 to S12**. Electronic transitions were simplified by displaying only those with the highest Natural Transition Orbitals (NTO) contributions, and the orbitals were colour-coded according to their character: PDI-π (purple), PDI-nitroso-centred (blue), PDI-carbonyl-centred (green), PPh_3_-σ (orange), PPh_3_-π (red). The corresponding absorption spectra are shown in **Figure S16** and **Figure S17**.

**Table S5:** Excitation energy (in nm), oscillator strength (f), and character in term of one electron excitation for the first 25 excitations of **IN1-a**. Orbitals have been coloured according to their character: PDI-π (purple), PDI-nitroso centred (blue), PDI-carbonyl centred (green).

| **IN1-a** | | | |
| --- | --- | --- | --- |
| Ex. state | λ (nm) | f (a.u.) | Character |
| 1 | 858 | 0.002 | \| 114 \| → \| 116 \| \| --- \| --- \| --- \| \| 114 \| → \| 117 \| |
| 2 | 546 | 0.665 | \| 115 \| → \| 116 \| \| --- \| --- \| --- \| |
| 3 | 472 | 0.046 | \| 114 \| → \| 116 \| \| --- \| --- \| --- \| \| 114 \| → \| 117 \| |
| 4 | 410 | 0.163 | \| 115 \| → \| 117 \| \| --- \| --- \| --- \| |
| 5 | 377 | 0.001 | \| 109 \| → \| 116 \| \| --- \| --- \| --- \| |
| 6 | 376 | 0.019 | \| 111 \| → \| 116 \| \| --- \| --- \| --- \| \| 113 \| → \| 116 \| |
| 7 | 365 | 0.025 | \| 111 \| → \| 116 \| \| --- \| --- \| --- \| \| 113 \| → \| 116 \| |
| 8 | 362 | 0.003 | \| 110 \| → \| 116 \| \| --- \| --- \| --- \| |
| 9 | 350 | 0.077 | \| 112 \| → \| 116 \| \| --- \| --- \| --- \| |
| 10 | 343 | 0.028 | \| 108 \| → \| 116 \| \| --- \| --- \| --- \| |
| 11 | 331 | 0.168 | \| 107 \| → \| 116 \| \| --- \| --- \| --- \| |
| 12 | 329 | 0.056 | \| 105 \| → \| 116 \| 187A \| \| --- \| --- \| --- \| --- \| |
| 13 | 324 | 0.018 | \| 106 \| → \| 116 \| \| --- \| --- \| --- \| \| 115 \| → \| 118 \| |
| 14 | 318 | 0.029 | \| 104 \| → \| 116 \| \| --- \| --- \| --- \| \| 106 \| → \| 116 \| |
| 15 | 317 | 0.016 | \| 104 \| → \| 116 \| \| --- \| --- \| --- \| \| 106 \| → \| 116 \| \| 115 \| → \| 118 \| |
| 16 | 311 | 0.014 | \| 115 \| → \| 119 \| \| --- \| --- \| --- \| |
| 17 | 307 | 0.007 | \| 114 \| → \| 118 \| \| --- \| --- \| --- \| |
| 18 | 297 | 0.000 | \| 110 \| → \| 117 \| \| --- \| --- \| --- \| |
| 19 | 287 | 0.023 | \| 113 \| → \| 117 \| \| --- \| --- \| --- \| |
| 20 | 285 | 0.053 | \| 111 \| → \| 117 \| \| --- \| --- \| --- \| \| 112 \| → \| 117 \| |
| 21 | 283 | 0.064 | \| 112 \| → \| 117 \| \| --- \| --- \| --- \| |
| 22 | 281 | 0.027 | \| 114 \| → \| 119 \| \| --- \| --- \| --- \| |
| 23 | 273 | 0.044 | \| 108 \| → \| 117 \| \| --- \| --- \| --- \| |
| 24 | 267 | 0.029 | \| 109 \| → \| 117 \| \| --- \| --- \| --- \| |
| 25 | 267 | 0.041 | \| 104 \| → \| 117 \| \| --- \| --- \| --- \| \| 109 \| → \| 117 \| \| 115 \| → \| 120 \| |

**Table S6:** Excitation energy (in nm), oscillator strength (f), and character in term of one electron excitation for the first 25 excitations of **IN1-b**. Orbitals have been coloured according to their character: PDI-π (purple), PDI-nitroso centred (blue), PDI-carbonyl centred (green).

| **IN1-b** | | | |
| --- | --- | --- | --- |
| Ex. state | λ (nm) | f (a.u.) | Character |
| 1 | 974 | 0.012 | \| 114 \| → \| 116 \| \| --- \| --- \| --- \| |
| 2 | 606 | 0.517 | \| 115 \| → \| 116 \| \| --- \| --- \| --- \| |
| 3 | 451 | 0.015 | \| 114 \| → \| 116 \| \| --- \| --- \| --- \| \| 114 \| → \| 117 \| \| 115 \| → \| 117 \| |
| 4 | 403 | 0.025 | \| 110 \| → \| 116 \| \| --- \| --- \| --- \| \| 113 \| → \| 116 \| |
| 5 | 392 | 0.159 | \| 113 \| → \| 116 \| \| --- \| --- \| --- \| \| 115 \| → \| 117 \| |
| 6 | 389 | 0.006 | \| 109 \| → \| 116 \| \| --- \| --- \| --- \| |
| 7 | 382 | 0.032 | \| 111 \| → \| 116 \| \| --- \| --- \| --- \| \| 112 \| → \| 116 \| |
| 8 | 379 | 0.015 | \| 110 \| → \| 116 \| \| --- \| --- \| --- \| \| 111 \| → \| 116 \| |
| 9 | 365 | 0.053 | \| 112 \| → \| 116 \| \| --- \| --- \| --- \| |
| 10 | 361 | 0.082 | \| 108 \| → \| 116 \| \| --- \| --- \| --- \| |
| 11 | 343 | 0.191 | \| 107 \| → \| 116 \| \| --- \| --- \| --- \| |
| 12 | 339 | 0.021 | \| 104 \| → \| 116 \|  \| \| --- \| --- \| --- \| --- \| \| 105 \| → \| 116 \| 187A \| |
| 13 | 336 | 0.053 | \| 106 \| → \| 116 \| \| --- \| --- \| --- \| |
| 14 | 333 | 0.030 | \| 115 \| → \| 118 \| \| --- \| --- \| --- \| |
| 15 | 330 | 0.014 | \| 104 \| → \| 116 \| \| --- \| --- \| --- \| \| 105 \| → \| 116 \| \| 115 \| → \| 118 \| |
| 16 | 316 | 0.024 | \| 115 \| → \| 119 \| \| --- \| --- \| --- \| |
| 17 | 305 | 0.003 | \| 114 \| → \| 118 \| \| --- \| --- \| --- \| |
| 18 | 285 | 0.000 | \| 110 \| → \| 117 \| \| --- \| --- \| --- \| \| 111 \| → \| 117 \| |
| 19 | 283 | 0.002 | \| 113 \| → \| 117 \| \| --- \| --- \| --- \| \| 114 \| → \| 119 \| \| 115 \| → \| 120 \| |
| 20 | 278 | 0.053 | \| 114 \| → \| 119 \| \| --- \| --- \| --- \| |
| 21 | 274 | 0.031 | \| 110 \| → \| 117 \| \| --- \| --- \| --- \| \| 112 \| → \| 117 \| |
| 22 | 270 | 0.000 | \| 109 \| → \| 117 \| \| --- \| --- \| --- \| |
| 23 | 270 | 0.010 | \| 112 \| → \| 117 \| \| --- \| --- \| --- \| \| 115 \| → \| 120 \| |
| 24 | 266 | 0.232 | \| 110 \| → \| 117 \| \| --- \| --- \| --- \| \| 111 \| → \| 117 \| \| 112 \| → \| 117 \| \| 115 \| → \| 120 \| |
| 25 | 265 | 0.058 | \| 108 \| → \| 117 \| \| --- \| --- \| --- \| |

**Table S7:** Excitation energy (in nm), oscillator strength (f), and character in term of one electron excitation for the first 25 excitations of **IN1-a-PPh_3_**. Orbitals have been coloured according to their character: PDI-π (purple), PDI-nitroso centred (blue), PDI-carbonyl centred (green), PPh_3_-σ (orange), PPh_3_- π (red).

| **IN1-a-PPh3** | | | |
| --- | --- | --- | --- |
| Ex. state | λ (nm) | f (a.u.) | Character |
| 1 | 840 | 0.001 | \| 182 \| → \| 185 \| \| --- \| --- \| --- \| \| 182 \| → \| 186 \| |
| 2 | 659 | 0.040 | \| 184 \| → \| 185 \| \| --- \| --- \| --- \| |
| 3 | 547 | 0.576 | \| 183 \| → \| 185 \| \| --- \| --- \| --- \| |
| 4 | 473 | 0.033 | \| 182 \| → \| 185 \| \| --- \| --- \| --- \| \| 182 \| → \| 186 \| |
| 5 | 452 | 0.006 | \| 184 \| → \| 186 \| \| --- \| --- \| --- \| |
| 6 | 435 | 0.016 | \| 181 \| → \| 185 \| \| --- \| --- \| --- \| |
| 7 | 420 | 0.009 | \| 180 \| → \| 185 \| \| --- \| --- \| --- \| |
| 8 | 408 | 0.120 | \| 183 \| → \| 186 \| \| --- \| --- \| --- \| |
| 9 | 393 | 0.011 | \| 179 \| → \| 185 \| \| --- \| --- \| --- \| |
| 10 | 387 | 0.002 | \| 178 \| → \| 185 \| \| --- \| --- \| --- \| |
| 11 | 373 | 0.018 | \| 174 \| → \| 185 \| \| --- \| --- \| --- \| \| 176 \| → \| 185 \| |
| 12 | 369 | 0.000 | \| 171 \| → \| 185 \|  \| \| --- \| --- \| --- \| --- \| |
| 13 | 368 | 0.002 | \| 177 \| → \| 185 \| \| --- \| --- \| --- \| |
| 14 | 364 | 0.051 | \| 176 \| → \| 185 \| \| --- \| --- \| --- \| |
| 15 | 356 | 0.001 | \| 173 \| → \| 185 \| \| --- \| --- \| --- \| |
| 16 | 352 | 0.048 | \| 175 \| → \| 185 \| \| --- \| --- \| --- \| |
| 17 | 345 | 0.020 | \| 172 \| → \| 185 \| \| --- \| --- \| --- \| |
| 18 | 337 | 0.004 | \| 184 \| → \| 187 \| \| --- \| --- \| --- \| |
| 19 | 334 | 0.012 | \| 170 \| → \| 185 \| \| --- \| --- \| --- \| |
| 20 | 328 | 0.005 | \| 181 \| → \| 186 \| \| --- \| --- \| --- \| |
| 21 | 325 | 0.096 | \| 169 \| → \| 185 \| \| --- \| --- \| --- \| |
| 22 | 324 | 0.041 | \| 166 \| → \| 185 \| \| --- \| --- \| --- \| \| 167 \| → \| 185 \| |
| 23 | 321 | 0.018 | \| 168 \| → \| 185 \| \| --- \| --- \| --- \| \| 183 \| → \| 187 \| |
| 24 | 319 | 0.018 | \| 180 \| → \| 186 \| \| --- \| --- \| --- \| |
| 25 | 316 | 0.027 | \| 168 \| → \| 185 \| \| --- \| --- \| --- \| |

**Table S8:** Excitation energy (in nm), oscillator strength (f), and character in term of one electron excitation for the first 25 excitations of **IN1-b-PPh_3_**. Orbitals have been coloured according to their character: PDI-π (purple), PDI-nitroso centred (blue), PDI-carbonyl centred (green), PPh_3_-σ (orange), PPh_3_- π (red).

| **IN1-b-PPh3** | | | |
| --- | --- | --- | --- |
| Ex. state | λ (nm) | f (a.u.) | Character |
| 1 | 904 | 0.012 | \| 182 \| → \| 185 \| \| --- \| --- \| --- \| |
| 2 | 669 | 0.121 | \| 183 \| → \| 185 \| \| --- \| --- \| --- \| \| 184 \| → \| 185 \| |
| 3 | 592 | 0.424 | \| 183 \| → \| 185 \| \| --- \| --- \| --- \| \| 184 \| → \| 185 \| |
| 4 | 455 | 0.012 | \| 182 \| → \| 185 \| \| --- \| --- \| --- \| \| 182 \| → \| 186 \| |
| 5 | 448 | 0.003 | \| 181 \| → \| 185 \| \| --- \| --- \| --- \| |
| 6 | 431 | 0.004 | \| 180 \| → \| 185 \| \| --- \| --- \| --- \| \| 181 \| → \| 185 \| |
| 7 | 417 | 0.077 | \| 184 \| → \| 186 \| \| --- \| --- \| --- \| |
| 8 | 402 | 0.014 | \| 179 \| → \| 185 \| \| --- \| --- \| --- \| |
| 9 | 398 | 0.016 | \| 178 \| → \| 185 \| \| --- \| --- \| --- \| |
| 10 | 395 | 0.039 | \| 179 \| → \| 185 \| \| --- \| --- \| --- \| \| 183 \| → \| 186 \| |
| 11 | 387 | 0.054 | \| 175 \| → \| 185 \| \| --- \| --- \| --- \| \| 176 \| → \| 185 \| \| 177 \| → \| 185 \| |
| 12 | 376 | 0.006 | \| 172 \| → \| 185 \|  \| \| --- \| --- \| --- \| --- \| |
| 13 | 375 | 0.003 | \| 177 \| → \| 185 \| \| --- \| --- \| --- \| |
| 14 | 372 | 0.071 | \| 174 \| → \| 185 \| \| --- \| --- \| --- \| \| 176 \| → \| 185 \| |
| 15 | 368 | 0.077 | \| 173 \| → \| 185 \| \| --- \| --- \| --- \| \| 175 \| → \| 185 \| |
| 16 | 367 | 0.030 | \| 173 \| → \| 185 \| \| --- \| --- \| --- \| |
| 17 | 354 | 0.002 | \| 171 \| → \| 185 \| \| --- \| --- \| --- \| \| 184 \| → \| 187 \| |
| 18 | 344 | 0.011 | \| 170 \| → \| 185 \| \| --- \| --- \| --- \| \| 171 \| → \| 185 \| |
| 19 | 336 | 0.023 | \| 183 \| → \| 187 \| \| --- \| --- \| --- \| \| 184 \| → \| 187 \| |
| 20 | 331 | 0.035 | \| 169 \| → \| 185 \| \| --- \| --- \| --- \| |
| 21 | 329 | 0.015 | \| 167 \| → \| 185 \| \| --- \| --- \| --- \| \| 183 \| → \| 187 \| |
| 22 | 328 | 0.018 | \| 166 \| → \| 185 \| \| --- \| --- \| --- \| \| 167 \| → \| 185 \| |
| 23 | 325 | 0.082 | \| 168 \| → \| 185 \| \| --- \| --- \| --- \| |
| 24 | 322 | 0.000 | \| 166 \| → \| 185 \| \| --- \| --- \| --- \| \| 167 \| → \| 185 \| |
| 25 | 318 | 0.017 | \| 184 \| → \| 188 \| \| --- \| --- \| --- \| |

**Table S9:** Excitation energy (in nm), oscillator strength (f), and character in term of one electron excitation for the first 25 excitations of **IN2p**. Orbitals have been coloured according to their character: PDI-π (purple), PDI-nitroso centred (blue), PDI-carbonyl centred (green), PPh_3_-σ (orange), PPh_3_- π (red).

| **IN2p** | | | |
| --- | --- | --- | --- |
| Ex. state | λ (nm) | f (a.u.) | Character |
| 1 | 579 | 0.017 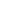 | \| 184 \| → \| 185 \| \| --- \| --- \| --- \| |
| 2 | 487 | 0.026 | \| 184 \| → \| 186 \| \| --- \| --- \| --- \| |
| 3 | 463 | 0.069 | \| 183 \| → \| 185 \| \| --- \| --- \| --- \| |
| 4 | 403 | 0.023 | \| 183 \| → \| 186 \| \| --- \| --- \| --- \| |
| 5 | 385 | 0.180 | \| 182 \| → \| 185 \| \| --- \| --- \| --- \| |
| 6 | 368 | 0.028 | \| 181 \| → \| 185 \| \| --- \| --- \| --- \| |
| 7 | 360 | 0.004 | \| 180 \| → \| 185 \| \| --- \| --- \| --- \| |
| 8 | 348 | 0.043 | \| 184 \| → \| 187 \| \| --- \| --- \| --- \| |
| 9 | 346 | 0.023 | \| 179 \| → \| 185 \| \| --- \| --- \| --- \| |
| 10 | 342 | 0.194 | \| 182 \| → \| 186 \| \| --- \| --- \| --- \| |
| 11 | 339 | 0.046 | \| 174 \| → \| 185 \| \| --- \| --- \| --- \| \| 182 \| → \| 186 \| \| 184 \| → \| 188 \| |
| 12 | 339 | 0.012 | \| 174 \| → \| 185 \|  \| \| --- \| --- \| --- \| --- \| |
| 13 | 330 | 0.024 | \| 178 \| → \| 185 \| \| --- \| --- \| --- \| |
| 14 | 327 | 0.016 | \| 176 \| → \| 185 \| \| --- \| --- \| --- \| \| 177 \| → \| 185 \| \| 178 \| → \| 185 \| |
| 15 | 322 | 0.003 | \| 175 \| → \| 185 \| \| --- \| --- \| --- \| |
| 16 | 320 | 0.008 | \| 177 \| → \| 185 \| \| --- \| --- \| --- \| |
| 17 | 318 | 0.009 | \| 172 \| → \| 186 \| \| --- \| --- \| --- \| |
| 18 | 317 | 0.025 | \| 180 \| → \| 186 \| \| --- \| --- \| --- \| \| 181 \| → \| 186 \| |
| 19 | 311 | 0.002 | \| 180 \| → \| 186 \| \| --- \| --- \| --- \| \| 181 \| → \| 186 \| |
| 20 | 304 | 0.003 | \| 183 \| → \| 187 \| \| --- \| --- \| --- \| |
| 21 | 302 | 0.046 | \| 173 \| → \| 185 \| \| --- \| --- \| --- \| |
| 22 | 301 | 0.030 | \| 168 \| → \| 185 \| \| --- \| --- \| --- \| |
| 23 | 298 | 0.010 | \| 168 \| → \| 185 \| \| --- \| --- \| --- \| \| 170 \| → \| 185 \| \| 171 \| → \| 185 \| \| 173 \| → \| 185 \| |
| 24 | 296 | 0.002 | \| 179 \| → \| 186 \| \| --- \| --- \| --- \| |
| 25 | 294 | 0.006 | \| 178 \| → \| 186 \| \| --- \| --- \| --- \| |

**Table S10:** Excitation energy (in nm), oscillator strength (f), and character in term of one electron excitation for the first 25 excitations of **IN3p**. Orbitals have been coloured according to their character: PDI-π (purple), PDI-nitroso centred (blue), PDI-carbonyl centred (green), PPh_3_-σ (orange), PPh_3_- π (red).

| **IN3p** | | | |
| --- | --- | --- | --- |
| Ex. state | λ (nm) | f (a.u.) | Character |
| 1 | 1115 | 0.097 | \| 184 \| → \| 185 \| \| --- \| --- \| --- \| |
| 2 | 546 | 0.036 | \| 184 \| → \| 186 \| \| --- \| --- \| --- \| |
| 3 | 510 | 0.149 | \| 184 \| → \| 187 \| \| --- \| --- \| --- \| |
| 4 | 499 | 0.055 | \| 184 \| → \| 188 \| \| --- \| --- \| --- \| |
| 5 | 487 | 0.024 | \| 184 \| → \| 189 \| \| --- \| --- \| --- \| |
| 6 | 455 | 0.003 | \| 184 \| → \| 190 \| \| --- \| --- \| --- \| |
| 7 | 417 | 0.227 | \| 183 \| → \| 185 \| \| --- \| --- \| --- \| |
| 8 | 406 | 0.147 | \| 184 \| → \| 191 \| \| --- \| --- \| --- \| |
| 9 | 381 | 0.016 | \| 184 \| → \| 193 \| \| --- \| --- \| --- \| |
| 10 | 371 | 0.005 | \| 184 \| → \| 192 \| \| --- \| --- \| --- \| |
| 11 | 365 | 0.106 | \| 182 \| → \| 185 \| \| --- \| --- \| --- \| |
| 12 | 352 | 0.008 | \| 184 \| → \| 194 \|  \| \| --- \| --- \| --- \| --- \| |
| 13 | 341 | 0.011 | \| 180 \| → \| 185 \| \| --- \| --- \| --- \| |
| 14 | 339 | 0.000 | \| 181 \| → \| 185 \| \| --- \| --- \| --- \| |
| 15 | 332 | 0.001 | \| 174 \| → \| 185 \| \| --- \| --- \| --- \| |
| 16 | 327 | 0.063 | \| 184 \| → \| 195 \| \| --- \| --- \| --- \| |
| 17 | 316 | 0.010 | \| 179 \| → \| 167 \| \| --- \| --- \| --- \| \| 184 \| → \| 183 \| |
| 18 | 311 | 0.003 | \| 178 \| → \| 185 \| \| --- \| --- \| --- \| |
| 19 | 309 | 0.014 | \| 179 \| → \| 185 \| \| --- \| --- \| --- \| \| 184 \| → \| 196 \| |
| 20 | 306 | 0.043 | \| 184 \| → \| 197 \| \| --- \| --- \| --- \| \| 184 \| → \| 198 \| |
| 21 | 302 | 0.056 | \| 177 \| → \| 185 \| \| --- \| --- \| --- \| \| 183 \| → \| 186 \| \| 184 \| → \| 197 \| |
| 22 | 300 | 0.018 | \| 176 \| → \| 185 \| \| --- \| --- \| --- \| \| 177 \| → \| 185 \| \| 183 \| → \| 186 \| |
| 23 | 299 | 0.008 | \| 176 \| → \| 185 \| \| --- \| --- \| --- \| |
| 24 | 296 | 0.016 | \| 167 \| → \| 185 \| \| --- \| --- \| --- \| \| 183 \| → \| 187 \| |
| 25 | 295 | 0.006 | \| 167 \| → \| 185 \| \| --- \| --- \| --- \| |

**Table S11:** Excitation energy (in nm), oscillator strength (f), and character in term of one electron excitation for the first 25 excitations of **IN6p**. Orbitals have been coloured according to their character: PDI-π (purple), PDI-nitroso centred (blue), PDI-carbonyl centred (green), PPh_3_-σ (orange), PPh_3_- π (red).

| **IN6p** | | | |
| --- | --- | --- | --- |
| Ex. state | λ (nm) | f (a.u.) | Character |
| 1 | 604 | 0.534 | \| 184 \| → \| 185 \| \| --- \| --- \| --- \| |
| 2 | 410 | 0.164 | \| 183 \| → \| 185 \| \| --- \| --- \| --- \| |
| 3 | 397 | 0.008 | \| 182 \| → \| 185 \| \| --- \| --- \| --- \| |
| 4 | 389 | 0.134 | \| 184 \| → \| 186 \| \| --- \| --- \| --- \| |
| 5 | 376 | 0.010 | \| 184 \| → \| 187 \| \| --- \| --- \| --- \| |
| 6 | 363 | 0.017 | \| 184 \| → \| 188 \| \| --- \| --- \| --- \| \| 184 \| → \| 189 \| |
| 7 | 350 | 0.003 | \| 181 \| → \| 185 \| \| --- \| --- \| --- \| \| 184 \| → \| 189 \| |
| 8 | 347 | 0.029 | \| 181 \| → \| 185 \| \| --- \| --- \| --- \| \| 184 \| → \| 188 \| \| 184 \| → \| 189 \| |
| 9 | 342 | 0.030 | \| 180 \| → \| 185 \| \| --- \| --- \| --- \| |
| 10 | 334 | 0.001 | \| 174 \| → \| 185 \| \| --- \| --- \| --- \| |
| 11 | 333 | 0.000 | \| 178 \| → \| 185 \| \| --- \| --- \| --- \| |
| 12 | 329 | 0.006 | \| 184 \| → \| 190 \|  \| \| --- \| --- \| --- \| --- \| |
| 13 | 321 | 0.018 | \| 179 \| → \| 185 \| \| --- \| --- \| --- \| |
| 14 | 314 | 0.010 | \| 177 \| → \| 185 \| \| --- \| --- \| --- \| |
| 15 | 309 | 0.018 | \| 184 \| → \| 191 \| \| --- \| --- \| --- \| \| 184 \| → \| 192 \| |
| 16 | 304 | 0.004 | \| 176 \| → \| 185 \| \| --- \| --- \| --- \| \| 184 \| → \| 191 \| |
| 17 | 303 | 0.004 | \| 176 \| → \| 185 \| \| --- \| --- \| --- \| \| 184 \| → \| 191 \| \| 184 \| → \| 192 \| |
| 18 | 297 | 0.050 | \| 173 \| → \| 185 \| \| --- \| --- \| --- \| \| 183 \| → \| 186 \| |
| 19 | 296 | 0.003 | \| 167 \| → \| 185 \| \| --- \| --- \| --- \| |
| 20 | 294 | 0.002 | \| 167 \| → \| 185 \| \| --- \| --- \| --- \| \| 168 \| → \| 185 \| |
| 21 | 293 | 0.014 | \| 175 \| → \| 185 \| \| --- \| --- \| --- \| |
| 22 | 292 | 0.039 | \| 173 \| → \| 185 \| \| --- \| --- \| --- \| \| 183 \| → \| 186 \| |
| 23 | 290 | 0.005 | \| 171 \| → \| 185 \| \| --- \| --- \| --- \| \| 172 \| → \| 185 \| \| 176 \| → \| 185 \| |
| 24 | 286 | 0.020 | \| 171 \| → \| 185 \| \| --- \| --- \| --- \| \| 172 \| → \| 185 \| \| 173 \| → \| 185 \| |
| 25 | 283 | 0.011 | \| 184 \| → \| 194 \| \| --- \| --- \| --- \| |

**Table S12:** Excitation energy (in nm), oscillator strength (f), and character in term of one electron excitation for the first 25 excitations of **PRp**. Orbitals have been coloured according to their character: PDI-π (purple), PDI-nitroso centred (blue), PDI-carbonyl centred (green), PPh_3_-σ (orange), PPh_3_- π (red).

| **PRp** | | | |
| --- | --- | --- | --- |
| Ex. state | λ (nm) | f (a.u.) | Character |
| 1 | 554 | 0.659 | \| 184 \| → \| 185 \| \| --- \| --- \| --- \| |
| 2 | 412 | 0.047 | \| 183 \| → \| 185 \| \| --- \| --- \| --- \| |
| 3 | 393 | 0.102 | \| 182 \| → \| 185 \| \| --- \| --- \| --- \| |
| 4 | 371 | 0.067 | \| 181 \| → \| 185 \| \| --- \| --- \| --- \| |
| 5 | 364 | 0.006 | \| 184 \| → \| 186 \| \| --- \| --- \| --- \| |
| 6 | 344 | 0.008 | \| 178 \| → \| 195 \| \| --- \| --- \| --- \| |
| 7 | 341 | 0.010 | \| 184 \| → \| 187 \| \| --- \| --- \| --- \| |
| 8 | 339 | 0.001 | \| 173 \| → \| 185 \| \| --- \| --- \| --- \| \| 174 \| → \| 185 \| |
| 9 | 338 | 0.003 | \| 172 \| → \| 185 \| \| --- \| --- \| --- \| \| 175 \| → \| 185 \| |
| 10 | 336 | 0.006 | \| 180 \| → \| 185 \| \| --- \| --- \| --- \| |
| 11 | 325 | 0.021 | \| 179 \| → \| 185 \| \| --- \| --- \| --- \| |
| 12 | 316 | 0.023 | \| 184 \| → \| 188 \|  \| \| --- \| --- \| --- \| --- \| |
| 13 | 312 | 0.002 | \| 177 \| → \| 185 \| \| --- \| --- \| --- \| |
| 14 | 309 | 0.002 | \| 174 \| → \| 185 \| \| --- \| --- \| --- \| |
| 15 | 307 | 0.002 | \| 176 \| → \| 185 \| \| --- \| --- \| --- \| |
| 16 | 303 | 0.004 | \| 168 \| → \| 185 \| \| --- \| --- \| --- \| \| 171 \| → \| 185 \| \| 172 \| → \| 185 \| \| 173 \| → \| 185 \| \| 184 \| → \| 189 \| |
| 17 | 303 | 0.001 | \| 184 \| → \| 189 \| \| --- \| --- \| --- \| |
| 18 | 301 | 0.071 | \| 171 \| → \| 185 \| \| --- \| --- \| --- \| |
| 19 | 300 | 0.003 | \| 167 \| → \| 185 \| \| --- \| --- \| --- \| \| 168 \| → \| 185 \| |
| 20 | 298 | 0.018 | \| 167 \| → \| 185 \| \| --- \| --- \| --- \| |
| 21 | 296 | 0.007 | \| 170 \| → \| 185 \| \| --- \| --- \| --- \| |
| 22 | 289 | 0.008 | \| 183 \| → \| 186 \| \| --- \| --- \| --- \| \| 184 \| → \| 190 \| |
| 23 | 287 | 0.020 | \| 184 \| → \| 190 \| \| --- \| --- \| --- \| \| 184 \| → \| 191 \| |
| 24 | 285 | 0.004 | \| 169 \| → \| 185 \| \| --- \| --- \| --- \| |
| 25 | 282 | 0.020 | \| 183 \| → \| 186 \| \| --- \| --- \| --- \| |

**Figure S16:** Computed spectra for **A)** **IN1-a**, **B) IN1-b, C) IN1a-PPh_3_, D) IN1-b-PPh_3_** reaction intermediates. 4-Nitrosophenanthrene has been used in the figure to simplify the PDI structure.

Comparison between the experimental and computed, convoluted spectra of **IN6p** and **PRp** (shown in the main text) reveals good agreement between both techniques and confirms the suitability of the chosen methodology. The first band, located at 600 nm, corresponds to a HOMO-LUMO π- π* transition, while the shoulder at 550 nm is predicted to have the same character, but arising from the **PRp** tautomer. The second band, located between 450 and 360 nm, is predicted to be composed by two transitions: a lower-energy HOMO-1 – LUMO transition centred at 410 nm, and a higher-energy HOMO – LUMO+1 transition at 389 nm. Spectra corresponding to each tautomer, along with the corresponding ones for **IN2p** and **IN3p** are depicted in **Figure S17**.

**Figure S17:** Computed spectra for **A)** **IN2-p-PPh3**, **B) IN3p, C) IN6p, D) PRp** reaction species. 4-Nitrosophenanthrene has been used in the figure to simplify the PDI structure.

### IV.4 Vertical excitations along scan coordinates

To study the behaviour of each excited state along the different reaction coordinates, TD-DFT calculations were carried out at each point of a relaxed scan performed on the ground state surface. The first 8 excited states were computed following the methodology described above, and a Natural Transition Orbital (NTO) analysis was performed for every transition to assess its character based on the shape and distribution of each NTO pair (donor and acceptor). In cases involving orbital mixing, and/or orbital evolution along the scan coordinate, the assignment was made while preserving the orbital character of each excitation and avoiding discontinuities in both energies and oscillator strengths. Each pair of orbitals contributing to a given transition was carefully compared with the corresponding pair from the previous scan point to ensure continuity throughout the reaction coordinate.

Notably, as shown in **Table S10,** during the P-H scan towards **IN3p**, most of the transitions undergo changes in orbital character, so that the assignment based on **IN1-a** excitations no longer accurately reflect the nature of the transitions. This aspect has been omitted from the main text to simplify the analysis of the photochemical behaviour of the system.

#### IV.4.1 IN1-a scan to IN1-b

The assignment of each electronic transition with its corresponding excitation level along the C-O bond distance scan between **IN1-**a and **IN1b** is depicted in **Table S13**. Scan was performed with a triphenylphosphine molecule.

**Table S13:** Excited state assignment along the C-O bond distance scan between **IN1-a** and **IN1-b** (distances in Ångstroms). Relevant excited states have been colored according to the main text.

|  | **IN1-a SCAN TO IN1-b** | | | | | | | | | | | |
| --- | --- | --- | --- | --- | --- | --- | --- | --- | --- | --- | --- | --- |
| **C-O Bond Distance:** | **3.98** | **3.88** | **3.77** | **3.62** | **3.49** | **3.36** | **3.25** | **3.15** | **3.06** | **2.98** | **2.91** | **2.8** |
| **Ex. State 1** | $1S_{NO}$ | $1S_{NO}$ | $1S_{NO}$ | $1S_{NO}$ | $1S_{NO}$ | $1S_{NO}$ | $1S_{NO}$ | $1S_{NO}$ | $1S_{NO}$ | $1S_{NO}$ | $1S_{NO}$ | $1S_{NO}$ |
| **Ex. State 2** | $1S_{CT}$ | $1S_{CT}$ | $1S_{CT}$ | $1S_{CT}$ | $1S_{CT}$ | $1S_{CT}$ | $1S_{CT}$ | $1S_{CT}$ | $1S_{CT}$ | $1S_{CT}$ | $1S_{CT}$ | $1S_{CT}$ |
| **Ex. State 3** | $1S_{\pi-\pi*}$ | $1S_{\pi-\pi*}$ | $1S_{\pi-\pi*}$ | $1S_{\pi-\pi*}$ | $1S_{\pi-\pi*}$ | $1S_{\pi-\pi*}$ | $1S_{\pi-\pi*}$ | $1S_{\pi-\pi*}$ | $1S_{\pi-\pi*}$ | $1S_{\pi-\pi*}$ | $1S_{\pi-\pi*}$ | $1S_{\pi-\pi*}$ |
| **Ex. State 4** | $1S_{NO-\pi*}$ | $2S_{CT}$ | $2S_{CT}$ | $2S_{CT}$ | $2S_{CT}$ | $2S_{CT}$ | $2S_{CT}$ | $2S_{CT}$ | $2S_{CT}$ | $1S_{NO-\pi*}$ | $1S_{NO-\pi*}$ | $1S_{NO-\pi*}$ |
| **Ex. State 5** | $2S_{CT}$ | $1S_{NO-\pi*}$ | $2S_{\pi-\pi*}$ | $2S_{\pi-\pi*}$ | $2S_{\pi-\pi*}$ | $2S_{\pi-\pi*}$ | $2S_{\pi-\pi*}$ | $2S_{\pi-\pi*}$ | $2S_{\pi-\pi*}$ | $2S_{CT}$ | $3S_{CT}$ | $3S_{CT}$ |
| **Ex. State 6** | $3S_{CT}$ | $3S_{CT}$ | $3S_{CT}$ | $3S_{CT}$ | $1S_{NO-\pi*}$ | $1S_{NO-\pi*}$ | $1S_{NO-\pi*}$ | $1S_{NO-\pi*}$ | $1S_{NO-\pi*}$ | $3S_{CT}$ | $2S_{CT}$ | $4S_{CT}$ |
| **Ex. State 7** | $4S_{CT}$ | $2S_{\pi-\pi*}$ | $1S_{NO-\pi*}$ | $1S_{NO-\pi*}$ | $3S_{CT}$ | $3S_{CT}$ | $3S_{CT}$ | $3S_{CT}$ | $3S_{CT}$ | $4S_{CT}$ | $4S_{CT}$ | $2S_{CT}$ |
| **Ex. State 8** | $2S_{\pi-\pi*}$ | $4S_{CT}$ | $4S_{CT}$ | $4S_{CT}$ | $4S_{CT}$ | $4S_{CT}$ | $4S_{CT}$ | $4S_{CT}$ | $4S_{CT}$ | $2S_{\pi-\pi*}$ | $2S_{\pi-\pi*}$ | $5S_{CT}$ |
| **Ex. State 9** | - | - | - | - | - | - | - | - | - | - | - | - |
| **Ex. State 10** | - | - | - | - | - | - | - | - | - | - | - | $2S_{\pi-\pi*}$ |

Relative energies of each excited state (according to the excitation order depicted in **Table S13**) along their oscillator strength is depicted in **Table S14**.

**Table S14:** Relative energy (in kcal/mol) and oscillator strengths (a.u.) of each excited state along the C-O bond distance scan between **IN1-a** and **IN1-b**.

| **C-O Bond Distance** | GS | $1S_{NO}$ | | $1S_{CT}$ | | $1S_{\pi-\pi*}$ | | $2S_{CT}$ | | $2S_{\pi-\pi*}$ | |
| --- | --- | --- | --- | --- | --- | --- | --- | --- | --- | --- | --- |
|  | ∆E | ∆E | O.S. | ∆E | O.S. | ∆E | O.S. | ∆E | O.S. | ∆E | O.S. |
| **3.99** | 2.4 | 36.0 | 0.0029 | 47.6 | 0.1146 | 53.9 | 0.1146 | 66.0 | 0.0891 | 71.1 | 0.0638 |
| **3.88** | 6.6 | 43.0 | 0.0063 | 51.9 | 0.0778 | 57.4 | 0.0778 | 69.5 | 0.0677 | 74.4 | 0.1319 |
| **3.77** | 8.5 | 45.0 | 0.0061 | 54.1 | 0.0533 | 58.9 | 0.0533 | 70.2 | 0.0146 | 72.3 | 0.2147 |
| **3.62** | 9.9 | 47.0 | 0.004 | 55.8 | 0.0442 | 60.9 | 0.0442 | 69.3 | 0.0414 | 71.9 | 0.2377 |
| **3.49** | 10.8 | 48.3 | 0.0016 | 57.5 | 0.0442 | 62.9 | 0.0442 | 68.1 | 0.0875 | 70.6 | 0.1891 |
| **3.36** | 11.2 | 48.9 | 0.0007 | 58.7 | 0.0442 | 65.0 | 0.0442 | 67.2 | 0.0772 | 69.1 | 0.0600 |
| **3.25** | 11.0 | 48.7 | 0.0020 | 58.5 | 0.042 | 64.9 | 0.0420 | 67.8 | 0.0361 | 69.1 | 0.0336 |
| **3.15** | 10.4 | 47.7 | 0.0048 | 57.2 | 0.0405 | 62.8 | 0.0405 | 69.3 | 0.0185 | 70.7 | 0.1365 |
| **3.06** | 9.5 | 46.1 | 0.0077 | 55.4 | 0.0483 | 60.4 | 0.0483 | 70.5 | 0.0199 | 72.0 | 0.1214 |
| **2.98** | 8.6 | 44.2 | 0.0098 | 53.6 | 0.0686 | 58.4 | 0.0686 | 73.0 | 0.0817 | 77.9 | 0.1258 |
| **2.91** | 7.9 | 42.3 | 0.0109 | 52.0 | 0.1026 | 57.0 | 0.1026 | 73.8 | 0.0843 | 78.7 | 0.1431 |

#### IV.4.2 IN1-b scan to IN2p

**Table S15:** Excited state assignment along the C-O bond distance scan between **IN1-b** and **IN2p** (distances in Ångstroms). Relevant excited states have been colored according to the main text.

|  | **IN1-b SCAN TO IN2p** | | | | | | | | | | | | | | | | |
| --- | --- | --- | --- | --- | --- | --- | --- | --- | --- | --- | --- | --- | --- | --- | --- | --- | --- |
| **C-O Dist.:** | | **2.80** | **2.70** | **2.60** | **2.50** | **2.40** | **2.30** | **2.20** | **2.10** | **2.00** | **1.90** | **1.80** | **1.70** | **1.60** | **1.50** | **1.40** |  |
| **Ex. St. 1** | | $1S_{NO}$ | $1S_{NO}$ | $1S_{NO}$ | $1S_{NO}$ | $1S_{NO}$ | $1S_{NO}$ | $1S_{NO}$ | $1S_{NO}$ | $1S_{NO}$ | $1S_{NO}$ | $1S_{NO}$ | $1S_{NO}$ | $1S_{NO}$ | $1S_{NO}$ | $1S_{NO}$ |  |
| **Ex. St. 2** | | $1S_{CT}$ | $1S_{CT}$ | $1S_{CT}$ | $1S_{CT}$ | $1S_{CT}$ | $1S_{CT}$ | $1S_{CT}$ | $1S_{CT}$ | $1S_{CT}$ | $1S_{CT}$ | $1S_{CT}$ | $1S_{CT}$ | $1S_{CT}$ | $1S_{CT}$ | $1S_{CT}$ |  |
| **Ex. St. 3** | | $1S_{\pi-\pi*}$ | $1S_{\pi-\pi*}$ | $1S_{\pi-\pi*}$ | $1S_{\pi-\pi*}$ | $1S_{\pi-\pi*}$ | $1S_{\pi-\pi*}$ | $1S_{\pi-\pi*}$ | $1S_{\pi-\pi*}$ | $1S_{\pi-\pi*}$ | $1S_{\pi-\pi*}$ | $1S_{\pi-\pi*}$ | $1S_{\pi-\pi*}$ | $1S_{\pi-\pi*}$ | $1S_{\pi-\pi*}$ | $1S_{\pi-\pi*}$ |  |
| **Ex. St. 4** | | $1S_{NO-\pi*}$ | $1S_{NO-\pi*}$ | $1S_{NO-\pi*}$ | $1S_{NO-\pi*}$ | $1S_{NO-\pi*}$ | $1S_{NO-\pi*}$ | $1S_{NO-\pi*}$ | $1S_{NO-\pi*}$ | $2S_{CT}$ | $2S_{CT}$ | $2S_{CT}$ | $2S_{CT}$ | $2S_{CT}$ | $2S_{CT}$ | $2S_{CT}$ |  |
| **Ex. St. 5** | | $3S_{CT}$ | $3S_{CT}$ | $3S_{CT}$ | $3S_{CT}$ | $3S_{CT}$ | $3S_{CT}$ | $3S_{CT}$ | $2S_{CT}$ | $1S_{NO-\pi*}$ | $1S_{NO-\pi*}$ | $2S_{\pi-\pi*}$ | $2S_{\pi-\pi*}$ | $2S_{\pi-\pi*}$ | $2S_{\pi-\pi*}$ | $2S_{\pi-\pi*}$ |  |
| **Ex. St. 6** | | $4S_{CT}$ | $4S_{CT}$ | $4S_{CT}$ | $4S_{CT}$ | $4S_{CT}$ | $4S_{CT}$ | $2S_{CT}$ | $3S_{CT}$ | $2S_{\pi-\pi*}$ | $2S_{\pi-\pi*}$ | $1S_{NO-\pi*}$ | $3S_{CT}$ | $3S_{CT}$ | $3S_{CT}$ | $3S_{CT}$ |  |
| **Ex. St. 7** | | $2S_{CT}$ | $2S_{CT}$ | $2S_{CT}$ | $5S_{CT}$ | $2S_{CT}$ | $2S_{CT}$ | $4S_{CT}$ | $4S_{CT}$ | $3S_{CT}$ | $3S_{CT}$ | $3S_{CT}$ | $1S_{NO-\pi*}$ | $4S_{CT}$ | $4S_{CT}$ | $4S_{CT}$ |  |
| **Ex. St. 8** | | $5S_{CT}$ | $5S_{CT}$ | $5S_{CT}$ | $2S_{CT}$ | $5S_{CT}$ | $5S_{CT}$ | $2S_{\pi-\pi*}$ | $2S_{\pi-\pi*}$ | $4S_{CT}$ | $4S_{CT}$ | $4S_{CT}$ | $4S_{CT}$ | $1S_{NO-\pi*}$ | $3S_{\pi-\pi*}$ | $3S_{\pi-\pi*}$ |  |
| **Ex. St. 9** | | - | - | - | - | - | $2S_{\pi-\pi*}$ | - | - | - | - | - | - | - | - | - |  |
| **Ex. St. 10** | | $2S_{\pi-\pi*}$ | $2S_{\pi-\pi*}$ | $2S_{\pi-\pi*}$ | $2S_{\pi-\pi*}$ | $2S_{\pi-\pi*}$ | - | - | - | - | - | - | - | - | $1S_{NO-\pi*}$ | - |  |

**Table S16:** Relative energy (in kcal/mol) and oscillator strengths (a.u.) of each excited state along the C-O bond distance scan between **IN1-b** and **IN2p**.

| **C-O Bond Distance** | GS | $1S_{NO}$ | | $1S_{CT}$ | | $1S_{\pi-\pi*}$ | | $2S_{CT}$ | | $2S_{\pi-\pi*}$ | |
| --- | --- | --- | --- | --- | --- | --- | --- | --- | --- | --- | --- |
|  | ∆E | ∆E | O.S. | ∆E | O.S. | ∆E | O.S. | ∆E | O.S. | ∆E | O.S. |
| **2.80** | 7.2 | 38.8 | 0.0116 | 50.0 | 0.1207 | 55.5 | 0.4239 | 75.8 | 0.0768 | 79.6 | 0.0392 |
| **2.70** | 7.4 | 37.9 | 0.0126 | 50.0 | 0.1232 | 55.5 | 0.4124 | 76.3 | 0.0771 | 79.7 | 0.0313 |
| **2.60** | 8.1 | 37.0 | 0.0137 | 50.4 | 0.1138 | 56.0 | 0.4057 | 77.5 | 0.0673 | 80.1 | 0.0188 |
| **2.50** | 9.3 | 36.6 | 0.0142 | 51.3 | 0.0828 | 57.3 | 0.4133 | 79.0 | 0.0532 | 81.2 | 0.0185 |
| **2.40** | 11.0 | 38.0 | 0.0140 | 53.5 | 0.0463 | 59.9 | 0.4116 | 80.3 | 0.0379 | 83.1 | 0.0477 |
| **2.30** | 13.1 | 40.8 | 0.014 | 56.9 | 0.027 | 63.0 | 0.366 | 81.5 | 0.0439 | 85.3 | 0.0697 |
| **2.20** | 15.4 | 44.0 | 0.0136 | 60.7 | 0.0152 | 66.0 | 2.99E-01 | 83.0 | 0.0832 | 86.1 | 0.1222 |
| **2.10** | 17.6 | 47.4 | 0.0123 | 64.5 | 0.0062 | 69.0 | 0.2282 | 84.7 | 0.092 | 88.5 | 0.1442 |
| **2.00** | 19.2 | 50.9 | 0.0095 | 68.2 | 0.0008 | 71.7 | 0.1598 | 83.0 | 0.0742 | 89.7 | 0.149 |
| **1.90** | 19.6 | 54.5 | 0.0055 | 71.2 | 0.0033 | 73.8 | 0.0968 | 83.7 | 0.0423 | 92.1 | 0.2443 |
| **1.80** | 18.0 | 58.0 | 0.0029 | 72.9 | 0.0156 | 74.2 | 0.0536 | 83.4 | 0.0284 | 89.6 | 0.1797 |
| **1.70** | 14.8 | 59.9 | 0.0042 | 73.4 | 0.0461 | 71.3 | 0.0252 | 82.0 | 0.0220 | 87.7 | 0.1840 |
| **1.60** | 11.1 | 59.8 | 0.0097 | 71.5 | 0.0605 | 68.6 | 0.021 | 80.0 | 0.0178 | 84.8 | 0.1855 |
| **1.50** | 8.6 | 58.6 | 0.0155 | 69.9 | 0.071 | 67.3 | 0.0181 | 78.9 | 0.0162 | 82.6 | 0.1854 |
| **1.40** | 9.6 | 59.5 | 0.0190 | 71.2 | 0.079 | 69.4 | 0.0152 | 80.8 | 0.0251 | 83.8 | 0.1667 |

Complete depiction of the evolution of the first eight excited states along the C-O bond distance coordinate is shown in **Figure S18**.

**Figure S18:** Complete depiction of the first eight vertical excitation energies along the relaxed C–O bond distance scan at the ground-state geometry, from **IN1-a** to **IN2p**. Yellow surfaces indicate the wavelengths accessible with the filters employed. The scanned bond distance is highlighted in red, and 4-nitrosophenanthrene is used in the figure to simplify the PDI structure.

#### IV.4.3 IN2p scan to IN3p

As introduced in the main text, due the nitroso cyclization, the character of the nitroso-centred MO becomes π in nature. Consequently, the labeling has been updated in the following tables.

**Table S17:** Excited state assignment along the P-H bond distance scan between **IN2p** and **IN3p** (distances in Ångstroms). Relevant excited states have been colored according to the main text.

|  | **IN2p SCAN TO IN3p** | | | | | | | | | | | | |
| --- | --- | --- | --- | --- | --- | --- | --- | --- | --- | --- | --- | --- | --- |
| **P-H Bond Distance:** | **2.66** | **2.56** | **2.46** | **2.36** | **2.26** | **2.16** | **2.06** | **1.96** | **1.86** | **1.76** | **1.66** | **1.56** | **1.46** |
| **Ex. State 1** | $1S_{\pi-\pi*}$ | $1S_{\pi-\pi*}$ | $1S_{\pi-\pi*}$ | $1S_{\pi-\pi*}$ | $1S_{\pi-\pi*}$ | $1S_{\pi-\pi*}$ | $1S_{\pi-\pi*}$ | $1S_{\pi-\pi*}$ | $1S_{\pi-\pi*}$ | $1S_{\pi-\pi*}$ | $1S_{\pi-\pi*}$ | $1S_{\pi-\pi*}$ | $1S_{\pi-\pi*}$ |
| **Ex. State 2** | $1S_{CT}$ | $1S_{CT}$ | $1S_{CT}$ | $1S_{CT}$ | $1S_{CT}$ | $1S_{CT}$ | $1S_{CT}$ | $1S_{CT}$ | $1S_{CT}$ | $1S_{CT}$ | $1S_{CT}$ | $1S_{CT}$ | $1S_{CT}$ |
| **Ex. State 3** | $2S_{\pi-\pi*}$ | $2S_{\pi-\pi*}$ | $2S_{\pi-\pi*}$ | $2S_{\pi-\pi*}$ | $2S_{\pi-\pi*}$ | $2S_{\pi-\pi*}$ | $2S_{\pi-\pi*}$ | $2S_{\pi-\pi*}$ | $4S_{\pi-\pi*}$ | $4S_{\pi-\pi*}$ | $4S_{\pi-\pi*}$ | $4S_{\pi-\pi*}$ | $4S_{\pi-\pi*}$ |
| **Ex. State 4** | $2S_{CT}$ | $2S_{CT}$ | $2S_{CT}$ | $3S_{\pi-\pi*}$ | $3S_{\pi-\pi*}$ | $3S_{\pi-\pi*}$ | $3S_{\pi-\pi*}$ | $3S_{\pi-\pi*}$ | $2S_{\pi-\pi*}$ | $5S_{\pi-\pi*}$ | $4S_{CT}$ | $4S_{CT}$ | $4S_{CT}$ |
| **Ex. State 5** | $3S_{\pi-\pi*}$ | $3S_{\pi-\pi*}$ | $3S_{\pi-\pi*}$ | $2S_{CT}$ | $2S_{CT}$ | $2S_{CT}$ | $4S_{\pi-\pi*}$ | $4S_{\pi-\pi*}$ | $5S_{\pi-\pi*}$ | $4S_{CT}$ | $5S_{\pi-\pi*}$ | $5S_{\pi-\pi*}$ | $5S_{\pi-\pi*}$ |
| **Ex. State 6** | $3S_{CT}$ | $3S_{CT}$ | $3S_{CT}$ | $3S_{CT}$ | $3S_{CT}$ | $4S_{\pi-\pi*}$ | $5S_{\pi-\pi*}$ | $5S_{\pi-\pi*}$ | $3S_{\pi-\pi*}$ | $5S_{CT}$ | $5S_{CT}$ | $5S_{CT}$ | $5S_{CT}$ |
| **Ex. State 7** | $4S_{CT}$ | $4S_{CT}$ | $4S_{CT}$ | $4S_{\pi-\pi*}$ | $4S_{\pi-\pi*}$ | $3S_{CT}$ | $2S_{CT}$ | $2S_{CT}$ | $3S_{CT}$ | $2S_{\pi-\pi*}$ | $2S_{\pi-\pi*}$ | $6S_{\pi-\pi*}$ | $6S_{\pi-\pi*}$ |
| **Ex. State 8** | $4S_{\pi-\pi*}$ | $4S_{\pi-\pi*}$ | $4S_{\pi-\pi*}$ | $4S_{CT}$ | $4S_{CT}$ | $5S_{\pi-\pi*}$ | $3S_{CT}$ | $3S_{CT}$ | $4S_{CT}$ | $6S_{\pi-\pi*}$ | $6S_{\pi-\pi*}$ | $2S_{\pi-\pi*}$ | $2S_{\pi-\pi*}$ |

**Table S18:** Relative energy (in kcal/mol) and oscillator strengths (a.u.) of each excited state along the P-H bond distance scan between **IN2p** and **IN3p**. Missing values are due to the fact that those excited states became out of the range of the first eight excited states computed.

| **P-H bond** | GS | $1S_{\pi-\pi*}$ | | $1S_{CT}$ | | $2S_{\pi-\pi*}$ | | $2S_{CT}$ | | $3S_{\pi-\pi*}$ | | $3S_{\pi-\pi*}$ | |
| --- | --- | --- | --- | --- | --- | --- | --- | --- | --- | --- | --- | --- | --- |
|  | ∆E | ∆E | O.S. | ∆E | O.S. | ∆E | O.S. | ∆E | O.S. | ∆E | O.S. | ∆E | O.S. |
| **2.66** | **8.5** | 57.8 | 0.0165 | 70.2 | 0.0689 | 67.1 | 0.0261 | 79.4 | 0.0230 | 82.7 | 0.1802 | 90.6 | 0.0434 |
| **2.56** | **8.7** | 57.1 | 0.0160 | 70.9 | 0.0652 | 66.7 | 0.0354 | 79.9 | 0.0324 | 83.0 | 0.1744 | 90.1 | 0.0799 |
| **2.46** | **9.0** | 56.3 | 0.0155 | 71.9 | 0.0622 | 66.3 | 0.0451 | 80.6 | 0.0476 | 83.7 | 0.1606 | 89.6 | 0.1119 |
| **2.36** | **9.6** | 55.6 | 0.0152 | 73.4 | 0.0592 | 65.9 | 0.0563 | 84.7 | 0.0670 | 81.5 | 0.1384 | 89.0 | 0.1230 |
| **2.26** | **10.5** | 55.0 | 0.0144 | 75.2 | 0.0606 | 65.7 | 0.0668 | 86.1 | 0.0988 | 82.6 | 0.0856 | 88.8 | 0.1969 |
| **2.16** | **11.8** | 54.5 | 0.0137 | 77.5 | 0.0631 | 65.8 | 0.0798 | 87.6 | 0.0144 | 84.0 | 0.0968 | 89.1 | 0.2687 |
| **2.06** | **13.5** | 54.0 | 0.0130 | 80.2 | 0.0675 | 66.0 | 0.0963 | 91.4 | 0.2085 | 85.6 | 0.0793 | 87.9 | 0.1222 |
| **1.96** | **15.6** | 53.5 | 0.0136 | 81.9 | 0.0640 | 67.1 | 0.1112 | 94.0 | 0.1912 | 86.4 | 0.0408 | 87.6 | 0.2025 |
| **1.86** | **17.9** | 52.1 | 0.0126 | 86.4 | 0.0770 | 68.1 | 0.1453 | - | - | 91.0 | 0.1802 | 84.9 | 0.0811 |
| **1.76** | **18.5** | 45.6 | 0.0669 | 88.4 | 0.3190 | 73.4 | 0.1419 | - | - | - | - | 76.1 | 0.1732 |
| **1.66** | **12.6** | 38.3 | 0.0790 | 81.4 | 0.1436 | 65.8 | 0.0461 | - | - | - | - | 68.4 | 0.1956 |
| **1.56** | **6.8** | 32.1 | 0.0816 | 76.5 | 0.2876 | 59.6 | 0.0230 | - | - | - | - | 62.0 | 0.1759 |
| **1.46** | **2.89** | **27.9** | 0.0827 | 72.5 | 0.3075 | 55.9 | 0.0095 | - | - | - | - | 57.9 | 0.1734 |

### IV.5 Excited state optimizations along scan coordinates

To obtain a more accurate picture of how the energies of the lowest excited states evolve during nitroso-group cyclization (**TS2p**), the most relevant excited states were optimized along the C-O bond coordinate. The results, summarised in **Figure S19**, shows that the $1S_{NO}$, $1S_{CT}$ and $1S_{\pi-\pi*}$ excited states exhibit higher reactions barriers than the ground state, consistent with the observation that populating the PDI LUMO does not favour cyclization. In contrast, $2S_{CT}$ state, in which the LUMO+1 orbital acts as the acceptor, displays an almost a barrierless behaviour for C-O bond formation. Unfortunately, all attempts to optimize the 2$S_{\pi-\pi*}$ state at various C-O bond distances were unsuccessful.

**Table S19:** Relative energy of each optimized excited state, along the C-O bond coordinate.

| GS | | $1S_{NO}$ | | $1S_{CT}$ | | $1S_{\pi-\pi*}$ | | $2S_{CT}$ | |
| --- | --- | --- | --- | --- | --- | --- | --- | --- | --- |
| R C-O (Å) | ∆E | R C-O (Å) | ∆E | R C-O (Å) | ∆E | R C-O (Å) | ∆E | R C-O (Å) | ∆E |
| **2.79** | 7.2 | **2.78** | 25.4 | **2.74** | 26.8 | **2.75** | 50.3 | **2.80** | 57.4 |
| **2.40** | 11.0 | **2.10** | 36.7 | **2.20** | 35.4 | **2.40** | 53.8 | **2.30** | 64.3 |
| **2.20** | 15.4 | **2.00** | 40.0 | **2.10** | 38.2 | **2.20** | 60.4 | **2.10** | 61.8 |
| **2.00** | 19.2 | **1.90** | 42.5 | **1.80** | 44.6 | **2.00** | 68.3 | **2.00** | 59.9 |
| **1.80** | 18.0 | **1.80** | 46.0 | **1.65** | 44.8 | **1.90** | 69.9 | **1.80** | 59.8 |
| **1.65** | 12.9 | **1.65** | 49.7 | **1.45** | 41.9 | **1.60** | 62.2 | **1.70** | 60.8 |
| **1.47** | 6.7 | **1.50** | 46.9 | **-** | - | **1.47** | 60.2 | **1.65** | 56.4 |
| **1.40** | 2.4 | **-** | - | **-** | - | **-** | - | **1.48** | 53.6 |

**Figure S19:** Optimized excited state evolution along the C-O bond distance between **IN1-b** and **IN2p**. Relative energies are referenced from **IN1-a** electronic energy. 4-Nitrosophenanthrene has been used in the figure to simplify the PDI structure.

### IV.6 Molecular Orbitals

Isocontour representations of the MOs mainly involved in the most characteristic transitions of each studied PDI-derivative are shown in **Figures S20-S27**.

**Figure S20:** Isocontour representation of the MOs involved in the most characteristic electronic transitions of **IN1-a** (isoval.=0.020 a.u.).

**Figure S21:** Isocontour representation of the MOs involved in the most characteristic electronic transitions of **IN1-b** (isoval.=0.020 a.u.).

**Figure S22:** Isocontour representation of the MOs involved in the most characteristic electronic transitions of **IN1-a-PPh_3_** (isoval.=0.020 a.u.).

**Figure S23:** Isocontour representation of the MOs involved in the most characteristic electronic transitions of **IN1-b-PPh_3_** (isoval.=0.020 a.u.).

**Figure S24**: Isocontour representation of the MOs involved in the most characteristic electronic transitions of **IN2p** (isoval.=0.020 a.u.).

**Figure S25:** Isocontour representation of the MOs involved in the most characteristic electronic transitions of **IN3p** (isoval.=0.020 a.u.).

**Figure S26:** Isocontour representation of the MOs involved in the most characteristic electronic transitions of **IN6p** (isoval.=0.020 a.u.).

**Figure S27:** Isocontour representation of the MOs involved in the most characteristic electronic transitions of **PRp** (isoval.=0.020 a.u.).

### IV.7 Electronic density difference across excited states

As discussed in the main text, optimization of the first two excited states of **IN2p** resulted in spontaneous deprotonation by the phosphine, accompanied by planarization of the PDI framework. Motivated by this behaviour, a vertical excitation study was performed along the relaxed P-H bond scan between **IN2p** and **IN3p**, (see **Figure 6** from the main text). The results show that for these two lowest excited states ($1S_{\pi-\pi*}$ and $1S_{CT}$), no barrier is observed for this transformation (the apparent barrier shown in **Figure 6** arises from orbital mixing with the $4S_{\pi-\pi*}$ state). The origin of this unusual behavior, which also involves the $4S_{\pi-\pi*}$ state, is not straightforward, as the orbitals involved in these transitions are all different. Consequently, the electron density difference between the ground state and each excited state were analyzed at the TS geometry to assess the influence of these transitions on the electronic density around the P-H-C triad. The results, shown in **Figure S28,** reveal that for the first two excited states and the $4S_{\pi-\pi*}$ state, photoexcitation redistributes electron density so that the C–H bond becomes more polarized and weaker, while phosphine becomes a more effective acceptor. This excited-state density pattern stabilizes the incipient C···H···P arrangement, removing the barrier for hydrogen transfer. This behavior is not observed for the $2S_{\pi-\pi*}$ and the $3S_{\pi-\pi*}$ states, where no increase in electron density is found on the hydrogen atom.

**Figure S28:** Electron density difference isocontours between the GS and the three lowest excited states of **IN2p** at the **TS2p** geometry (isoval. = 0.001 a.u.).

1. Hendsbee, A.D., Sun, J.-P., Law, W.K., Yan, H., Hill, I.G., Spasyuk, D.M, Welch, G.C., *Chem. Mater.* **2016**, *28*, 7098-7109. [↑](#footnote-ref-1)
2. El-Berjawi, R.; Hudhomme, P., *Dyes and Pigments* **2018,** *159*, 551-556. [↑](#footnote-ref-2)
3. Roger, M.; Krupka, O.; Alévêque, O.; Levillain E.; Hudhomme , P., *Org. Chem. Front*. **2024**, *11*, 7069-7077. [↑](#footnote-ref-3)
